# Relationship between Pesticide Standards for Classification of Water Bodies and Ecotoxicity: A Case Study of the Brazilian Directive

**DOI:** 10.3390/toxics10120767

**Published:** 2022-12-08

**Authors:** Esmeralda Pereira de Araújo, Eloisa Dutra Caldas, Eduardo Cyrino Oliveira-Filho

**Affiliations:** 1 Faculty of Planaltina—FUP, University of Brasilia—UnB, Federal District, Planaltina 73345-010, Brazil; 2 Toxicology Laboratory, Faculty of Health Sciences, University of Brasília—UnB, Federal District, Brasília 70910-900, Brazil; 3 Brazilian Agricultural ReseArch. Corporation—Embrapa Cerrados, Federal District, Planaltina 73310-970, Brazil

**Keywords:** environmental toxicology, pesticide limits, water bodies, water quality

## Abstract

The objective of this study was to evaluate if the maximum values (MVs) for pesticides in surface freshwater included in CONAMA directive 357/2005 are safe for aquatic biota, comparing them with ecotoxicology data published in the literature. The terms “aquatic toxicity”, “chronic” “acute”, “LC_50_”, “EC_50_”, “NOEL”, “NOEC” and the name of each pesticide were used for searches on the research platforms. Data from 534 tests reported in 37 published articles and three ecotoxicological databases were included in this study; 24% of the tests were carried out with producer organisms, 34% with primary consumers and 42% with secondary consumers. Microcrustaceans of the *Daphnia* genus and the fishes *Pimephales promelas* and *Oncorhynchus mykiss* were the organisms most used. Atrazine, alachlor and metolachlor were the most investigated pesticides. Atrazine and alachlor are approved in Brazil, with atrazine ranking fourth among the most used pesticides in the country. The results indicated that of the 27 pesticides included in the standard directive, 17 have a risk quotient (RQ) higher than the level of concern for at least one ecotoxicological parameter and may not protect the aquatic biota. The insecticide malathion, used in various agricultural crops in Brazil, was the one that presented the highest RQs (3125 and 3,125,000 for freshwaters classified as 1/2 and 3, respectively), related to a LC_50_ (96 h) of 0.000032 µg/L in *Chironomus ramosus*. The results indicate that CONAMA directive 357/2005 should be updated in line with the current use of pesticides in the country, and the MVs should be re-evaluated so that they do not represent toxicity for the aquatic biota.

## 1. Introduction

The environmental behavior of pesticides, especially in relation to their transport and stability in water bodies, can have impact on human health and on the environment [1,2]. Toxic pesticide levels in aquatic systems may eliminate aquatic species, reduce biodiversity and compromise the functioning of ecosystems [3]. Aiming to provide protection, the regulatory jurisdictions of different countries establish limit values for pesticides in matrices such as soil, drinking water and agricultural commodities [4]. However, the regulation of these contaminants in surface freshwater is still limited in most countries [5]. Pesticide standards for surface freshwater are necessary in order to maintain the protection of the aquatic ecosystem and of human health against possible toxicological effects.

Directive 2013/39/EU of the European Union establishes environmental quality standards (EQS) for priority substances and other pollutants, including pesticides, in surface water, and it also establishes biota EQS for substances that are not very soluble in water and that accumulate in the organisms [6]. In the United States, the EPA’s Office of Pesticide Programs establishes Aquatic Life Benchmarks that are specific for each biota [7]. In Brazil, CONAMA directive No. 357, from 17 MArch. 2005, determines the quality parameters, including the establishment of maximum values (MV) for pesticides in surface freshwater classes 1/2 and 3, which are destined for multiple uses (Table S1). Classes 1 and 2 can be destined for the protection of aquatic biota, but this may not be their predominant use [8]. For example, class 2 water can also be used for supplying human consumption, primary-contact recreation, aquiculture and fishing. Even so, in accordance with the Directive and independently of its uses, class 2 water may not have characteristics that represent a chronic toxic effect on the biota. On the other hand, class 3 water does not include protection of aquatic biota among its uses, although the water within this class cannot exert an acute toxic effect on organisms. However, there are few water bodies that have been approved for inclusion in a determined class [9], and in this situation all freshwater is considered class 2 [8]. In other words, most Brazilian surface freshwater is class 2.

The toxic effects of pesticides on biota are evaluated in Brazil during the registration of new pesticides, using the data from ecotoxicological tests with non-target organisms [10], which could fit in a prospective approach, pre-registration [3]. Environmental data are used during the re-evaluation of the pesticide, which could be considered a retrospective approach. For aquatic organisms, the Brazilian Institute of the Environment and Renewable Natural Resources (IBAMA) requests studies with algae, microcrustaceans and fish, and the results are extrapolated for use in the whole taxon. Considering the trophic levels, some of the model organisms used in tests are the algae *Raphidocelis subcapitata* and *Scenedesmus subspicatus* (producer organisms), the microcrustaceans of the *Daphnia* genus (primary consumer) and the fish *Danio rerio* (secondary consumer) [11]. 

Although ecotoxicological studies are carried out under laboratory-controlled conditions and may not reflect the biotic and abiotic conditions in aquatic ecosystems [12,13], they are used to derive concentration levels that are safe or can cause toxicity for the biota. The studies have acceptable levels of uncertainty, and are used in the decisions making process by some regulatory agencies [13,14,15]. However, this is not the case in CONAMA directive 357/2005, which does not consider the evaluation conducted by IBAMA. Furthermore, the basis for the establishment of MVs and how the compounds were selected are not publicly available [16].

Bearing in mind that the use of pesticides has grown in Brazil, as well as worldwide [17,18], and that the number of authorized substances has also increased in the country [19], it is important to consider the potential impact on the aquatic biota arising from the use of these products. In addition, the presence in water of organochloride pesticides that are already banned in most countries (persistent organic pollutants, POPs) can also represent a toxic effect on aquatic organisms. Thus, the objective of this study was to evaluate if the maximum values (MVs) for pesticides in surface freshwater found in the Brazilian regulations (CONAMA directive 357/2005) are safe for aquatic biota, comparing them with ecotoxicology data published in the literature to calculate risk quotients (RQ).

## 2. Materials and Methods

In order to carry out this study, reseArch. was done in the Web of Science, Scopus and Google Scholar databases, using the descriptors “aquatic toxicity”, “chronic” “acute”, “LC_50_”, “EC_50_”, “NOEL”, “NOEC” and the name of each pesticide listed in Table 1. Selection criteria were studies conducted with surface freshwater aquatic organisms and pesticides included in the CONAMA directive. Additionally, data on the ecotoxicity of these substances were searched in the Pesticide Properties Database [20], NORMAN Ecotoxicology Database [21] and Aquatic Life Benchmarks [7], which cover a large range of organisms and pesticides and have been used by other authors [22,23]. 

To evaluate whether the maximum pesticide values in surface water (MV) established by CONAMA Directive 357/2005 are safe for aquatic organisms, the risk quotient (RQ) for each pesticide was estimated by dividing its MV by the relevant toxicological endpoint (chronic or acute) (RQ = ML/endpoint) [25]. The endpoints to estimate the acute risk were LC_50_ (lethal concentration) and EC_50_ (effective concentration); the endpoints to estimate the chronic risk were LOEC (lowest observed effect concentration), NOAEC (no observed adverse effect concentration), NOEC (no observed effect concentration), LOEC (lowest observed effect concentrations), PNOEC (predicted no effect concentration) or MATC (maximum acceptable toxicant concentrations). Risk may exist when the RQ is higher than the Level of Concern (LOC) as established by the EPA [25], which is 0.5 for acute high risk and 1 for chronic risk to aquatic animals, and 1 for acute risk to plants.

The organisms used in the tests were classified according to trophic levels (producer, primary consumer and secondary consumer) in the aquatic ecosystem. The aquatic organisms most used in the tests were also identified, as well the quantitative measure of tests carried out for each pesticide. 

## 3. Results and Discussion

The data included in this study were obtained from 37 papers [26,27,28,29,30,31,32,33,34,35,36,37,38,39,40,41,42,43,44,45,46,47,48,49,50,51,52,53,54,55,56,57,58,59,60,61,62] and three databases [7,20,21] (Supplementary Material). The papers were retrieved from 20 scientific journals, mainly Environmental Contamination Toxicology and Chemistry (six papers) and Ecotoxicology and Environmental Safety (five papers) and were published in the period of 1981 to 2021. The number of journals in the first quartile of quality were: three of the 18 journals in Web of Science database, eight out of 19 journals in Scopus. Journals retrieved from Google Scholar have h5 index ranging from 12 (Annales de Limnologie-International Journal of Limnology) to 225 (Science of the Total Environment). All the studies were conducted in a laboratory setting, but this information is not included in the three databases, which are updated online. 

Out of the total of 534 tests with aquatic organisms included in the studies, 24% were carried out with producing organisms, 34% with primary consumers and 42% with secondary consumers. The producers form the base of the aquatic food chain and are food for the primary consumers, which play an important cycling role in the environment and are food for the secondary consumers, which are the vertebrate organisms that form the aquatic ecosystem [11]. To confirm the toxic effect of a substance for regulatory purposes, it is recommended that an evaluation be carried out of with at least three species that represent the aquatic ecosystem, and they should ideally come from different trophic levels of the food chain [11,63]. However, many studies are not done for a regulatory purpose, and some evaluated the toxicity of one or more pesticides towards only one species [31,40,41,47,48]. Very few studies, however, are conducted with species that are representatives of the Brazilian ecosystems. 

**Figure 1:**
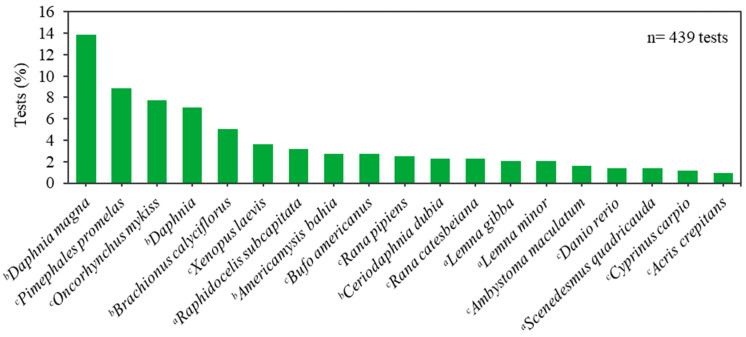
Freshwater species most used in the ecotoxicological studies with pesticides included in CONAMA standard directive 357/05. ^a^ Producer organism; ^b^ Primary consumer; ^c^ Secondary consumer.

From the set of 534 tests, 82% (439) classified the organisms at genus or species level. Some species from the genera *Najas* sp. and *Anabaena* sp., and 14 other species (10 producers, 2 primary consumers and 2 secondary consumers) are native in Brazil [64,65,66,67]. About 21% of the studies used the genus *Daphnia* and the species *Daphnia magna* (crustaceans), indicating that this group is the model most often used, followed by the fish species *Pimephales promelas* (9%) and *Oncorhynchus mykiss* (8%) (Figure 1). A review of European laboratory protocols for the ecotoxicity of systemic pesticides and microbial toxins in genetically modified plants also found these organisms as the most often considered in the directives [68].

**Figure 2:**
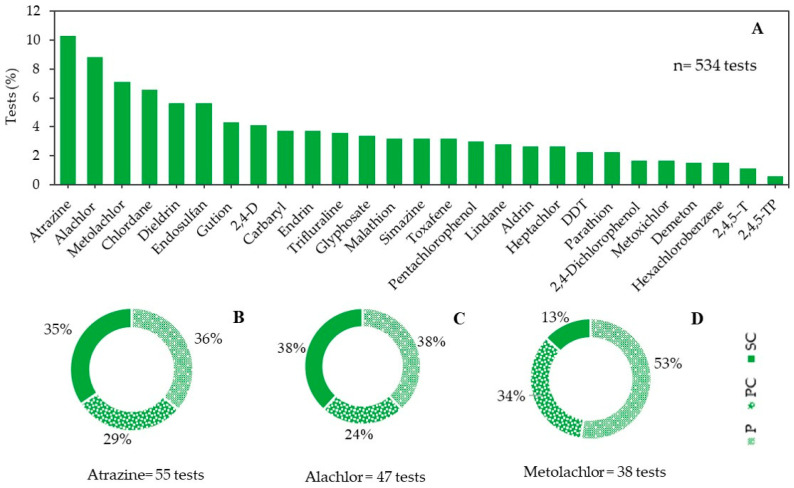
(**A**) Percentage of tests conducted with the pesticides listed in the tests shown in CONAMA standard directive 357/05. (**B**–**D**) Percentage of tests conducted with the pesticides in producer (P), primary consumer (PC) and secondary consumer organisms (SC).

Figure 2 shows that most of the ecotoxicity tests were conducted with atrazine (10%; the majority with producers), alachlor (9%; the majority with producers and secondary consumers) and metolachlor (7%; the majority with producers). In a review of 146 studies on pesticides in surface freshwater, Araújo et al. [5] showed that, historically, these pesticides are among the most investigated in water worldwide, and that in general atrazine was also the active ingredient detected at the highest concentrations. Atrazine, the fourth most sold active ingredient in Brazil [69], and alachlor are registered in the country for pre- and/or post-emergence use in a variety of crops [24], while the use of metolachlor was prohibited in 2010 [70]. Table 1 shows that the environmental classification for the pesticides approved in Brazil varies from I (extremely hazardous) for atrazine, 2,4-D, glyphosate, malathion and trifluraline, to IV (slightly hazardous) for malathion, depending on the product formulation [19]. It should be noted that, in addition to atrazine and alachlor, only six of the 27 pesticides included in CONAMA 357/2005 are still approved for use in the country (carbaryl, 2,4-D, glyphosate, malathion, simazine and trifluraline), and 12 are considered POPs (Table 1).

Table 2 shows the 17 pesticides that have a RQ higher than the LOC for at least one organism tested, indicating that the biota may not be protected when present in an aquatic environment with concentrations at the legal levels. Although the MVs in the directive for water quality in Brazil were established to classify different water uses (classes) and not specifically for the protection of the biota, the results of this study indicate that these levels should be reviewed.

Considering the trophic levels, the group of secondary consumers is the one that shows a RQ higher than 1 (Table 2). This result may have arisen because the representatives of the genus *Daphnia* (crustaceans) were the organisms most used in tests (Figure 1). Indeed, the pesticides that presented the greatest toxicity were the insecticides malathion and endosulfan, with LC_50_ (96 h) of 0.000032 (RQ of 3,125,000 for class 3 water) and 0.00036 µg/L (RQ of 611), respectively, for the larvae of the aquatic insect *Chironomus ramosus* [52]. Malathion has also the highest chronic RQ for invertebrates and *Daphnia magna* (1666.7; Table 2). 

**Table 2:** Pesticides listed in CONAMA directive 357/05, for which the risk quotient is higher than the level of concern (LOC) for at least one tested organism. LOC = 0.5 for acute risk to aquatic animals; LOC = 1 for chronic risk to aquatic animals and 1 for acute risk to plants [7].

Pesticide	Risk Quotient Class 1,2/3 (µg/L)	Endpoint: Concentration (µg/L)	Tested Organism	Reference
Alachlor	3/-	EC_50_ (72 h): 6.69	*Raphidocelis subcapitata* ^a^	[26]
	2/-	EC_50_ (96 h): 10	*Raphidocelis subcapitata* ^a^	[27]
	2/-	EC_50_ (7 d)-biomass: 10	*Lemna minor* ^a^	[20]
	12.2/-	EC_50_ (<10 d): 1.64	Nonvascular plants ^a^	[7]
	8.7/-	EC_50_ (<10 d): 2.3	Vascular plants ^a^	[7]
Aldrin	-/3	NOEC-ratio of ovigerous to non-ovigerous females: 0.01	*Brachionus calyciflorus* ^b^	[31]
	-/1.8	LC_50_ (96 h): 0.017	*Pimephales promelas* ^c^	[21]
Dieldrin	5/30	LOEC-population growth rate: 0.001	*Brachionus calyciflorus* ^b^	[31]
	5/30	NOEC-ratio of ovigerous to non-ovigerous females: 0.001	*Brachionus calyciflorus* ^b^	[31]
	-/3	LOEC-ratio of ovigerous to non-ovigerous females: 0.01	*Brachionus calyciflorus* ^b^	[31]
Atrazine	2/2	EC_50_ (<10 d): <1	Nonvascular plants ^a^	[7]
Carbaryl	-/1.2	NOEC-resting egg production: 60	*Brachionus calyciflorus* ^b^	[37]
	-/3.5	NOEC-resting egg hatching rate: 20	*Brachionus calyciflorus* ^b^	[37]
	-/1.2	LOEC-resting egg hatching rate: 60	*Brachionus calyciflorus* ^b^	[37]
	-/41.2	EC_50_ or LC_50_ (48 or 96 h): 1.7	Invertebrates ^b^	[7]
	-/140	NOAEC: 0.5	Invertebrates ^b^	[7]
	-/10.9	EC_50_ (48 h): 6.4	*Daphnia pulex* ^b^	[20]
	-/12.3	LC_50_ (96 h): 5.7	*Americamysis bahia* ^b^	[20]
	-/11.7	NOAEC: 6	Fish ^c^	[7]
Chlordane	-/2.4	LC_50_ (96 h): 0.127	*Neocaridina denticulate* ^b^	[43]
	-/1.7	NOEC (14 d)-survival: 0.18	*Ceriodaphnia dubia* ^b^	[44]
	-/1.7	NOEC (14 d)- number of offspring per female: 0.18	*Ceriodaphnia dubia* ^b^	[44]
	-/1.7	NOEC (21 d)- number of offspring per female: 0.18	*Daphnia magna* ^b^	[44]
	-/4.3	LC_50_ (48 h)-trans: 0.07	*Daphnia* ^b^	[21]
	-/7.5	LC_50_ (96 h)-trans: 0.04	*Pimephales promelas* ^c^	[21]
2,4-D	-/1	LOEC: 29	*Hyalella meinerti* ^b^	[48]
	-/1	NOEC: <29	*Hyalella meinerti* ^b^	[48]
	1.2/9.3	LC_50_ (48 h): 3.22	*Daphnia* ^b^	[21]
	1.5/11.6	LC_50_ (96 h): 2.59	*Pimephales promelas* ^c^	[21]
Demeton	-/1.3	EC_50_ (48 h) ^d^: 10.4	*Daphnia pulex* ^b^	[20]
	-/1.6	LC_50_ (48 h) ^d1^: 8.62	*Daphnia* ^b^	[21]
	-/3.2	LC_50_ (96 h) ^d1^: 4.43	*Pimephales promelas* ^c^	[21]
	-/3.2	LC_50_ (48 h) ^d2^: 4.44	*Daphnia* ^b^	[21]
DDT	-/1	EC_50_ (48 h) ^e^: 1	*Bosmina longirostris* ^b^	[20]
Endosulfan	5.6/22	NOAEC: 0.01	Invertebrates ^b^	[7]
	0.6/2.2	LC_50_ (96 h): 0.1	Fish ^c^	[7]
	2.4/9.6	NOAEC: 0.023	Fish ^c^	[7]
	155.6/611.1	LC_50_ (96 h): 0.00036	*Chironomus ramosus ^b^*	[52]
	112/440	NOEC (28 d): 0.0005	*Cyprinodon variegatus* ^c^	[20]
Endrin	-/1.1	LC_50_ (48 h): 0.19	*Daphnia* ^b^	[21]
	2/100	LC_50_ (96 h): 0.002	*Pimephales promelas* ^c^	[21]
	-/1.7	NOEC (21 d): 0.12	*Cyprinodon variegatus* ^c^	[20]
Glyphosate	5.4/23.3	EC_50_ (7 d): 12	*Lemna gibba* ^a^	[20]
Lindane	-/2	EC_50_ or LC_50_ (48 or 96 h): 1	*Invertebrates* ^b^	[7]
	-/1.2	LC_50_ (96 h): 1.7	Fish ^c^	[7]
	-/0.7	LC_50_ (96 h): 2.9	*Oncorhynchus mykiss* ^c^	[20]
Malathion	1/1020.4	EC_50_ or LC_50_ (48 or 96 h): 0.098	Invertebrates ^b^	[7]
	1.7/1666.7	NOAEC: 0.06	Invertebrates ^b^	[7]
	-/111.1	LC_50_ (48 h): 0.9	*Daphnia magna* ^b^	[57]
	-/4.9	LC_50_ (48 h): 20.32	*Daphnia* ^b^	[21]
	-/142.9	EC_50_ (48 h): 0.7	*Daphnia magna* ^b^	[20]
	1.7/1666.7	NOEC (21 d): 0.06	*Daphnia magna* ^b^	[20]
	-/66.7	LC_50_ (96 h): 1.5	*Americamysis bahia* ^b^	[20]
	-/24.4	LC_50_ (96 h): 4.1	Fish ^c^	[7]
	-/11.6	NOAEC: 8.6	Fish ^c^	[7]
	3125/3,125,000	LC_50_ (96 h): 0.000032	*Chironomus ramosus* ^b^	[52]
	-/22.3	LC_50_ (96 h): 4.48	*Pimephales promelas* ^c^	[21]
	-/5.6	LC_50_ (96 h): 18	*Oncorhynchus mykiss* ^c^	[20]
	-/1.1	NOEC (21 d): 91	*Oncorhynchus mykiss* ^c^	[20]
Metolachlor	1.3/-	EC_50_ (<10 d): 8	Nonvascular Plants ^a^	[7]
	10/-	NOAEC: 1	Invertebrates ^b^	[7]
Metoxichlor	-/0.7	LC_50_ (48 h): 30	*Daphnia* ^b^	[21]
	-/14.3	EC_50_ or LC_50_ (48 or 96 h): 1.4	Invertebrates ^b^	[7]
	-/25.6	EC_50_ (48 h): 0.78	*Daphnia magna* ^b^	[20]
	-/20	NOEC (21 d): 1	*Daphnia magna* ^b^	[20]
	-/1.3	LC_50_ (96 h): 15	Fish ^c^	[7]
Parathion	-/92.1	LC_50_ (48 h): 0.38	*Daphnia magna* ^b^	[57]
	-/46.7	LC_50_ (48 h): 0.75	*Daphnia* ^b^	[21]
	-/14	EC_50_ (48 h): 2.5	*Daphnia magna* ^b^	[20]
	-/350	NOEC (21 d): 0.1	*Daphnia magna* ^b^	[20]
	-/318.2	LC_50_ (96 h): 0.11	*Americamysis bahia* ^b^	[20]

d: day; h: hour; LC_50_: lethal concentration; EC_50_: effective concentration; LOEC: lowest observed effect concentration; NOAEC: no observed adverse effect concentration; NOEC: no observed effect concentration; LOEC: lowest observed effect concentrations. ^a^ Producer organism; ^b^ Primary consumer; ^c^ Secondary consumer; ^d^ Demeton; ^d1^ Isomer S; ^d2^ Isomer O; ^e^ Degradation product of DDE. All ecotoxicological studies were conducted in a laboratory setting, except for Refs. [7,20,21], where this information was not available.

Malathion was the seventh most commonly sold pesticide in Brazil in 2020 (15,702.11 ton) [69] and is registered for use on 23 crops, including vegetables, fruits and cereals [24]. Due to its persistence in the environment, the organochlorine endosulfan was prohibited in countries that are signatories of the Stockholm Convention, is classified as a POP [71] and its monitoring in water bodies still takes place in many countries [8,72]. In directive 2013/39/EU of the European Union, the endosulfan annual average are 0.005 and 0.0005 µg/L for inland surface waters and other surface waters, respectively, and the maximum allowable concentration are 0.01 and 0.004 µg/L, respectively [6]. These limits are more restrictive than the CONAMA 357/2005 MVs, but are still above the LC_50_ for *Chironomus ramosus* larvae (Table 2).

Various studies evaluated the levels of pesticides in surface freshwaters in Brazilian states, finding maximum concentrations that were equal to or lower than the MV established by the CONAMA directive (Table 1), with one exception (2,4-D for class 1/2). Pires et al. [73] detected glyphosate (2.3 μg/L) in Pará, Severo et al. [74] found atrazine (2 μg/L) and 2,4-D (30 μg/L, MV of 4 μg/L) in Rio Grande do Sul, Souza et al. [75] found atrazine (0.26 μg/L), Vieira et al. [76] detected atrazine (0.2 μg/L) and malathion (0.05 μg/L) in Paraná and Machado et al. [77] confirmed the occurrence of atrazine (0.32 μg/L) in São Paulo. However, the maximum concentrations detected for atrazine, 2,4-D, and malathion [74,75,76,77] are higher than the ecotoxicological parameters included in this study (Table 2) and may represent a toxic effect on the biota. This also shows the importance of considering the data on ecotoxicity in the Brazilian legislation for pesticides in surface water. 

The need for a legislation revision identified in this study is corroborated by Brovini et al. [78] using monitoring data and the RQ approach. According to the authors, although most of the environmental concentrations were below the MLs, they were enough to pose a high risk for the aquatic ecosystems. In addition, using monitoring data, Albuquerque et al. [16] observed the potential risk to aquatic life for 59% of the pesticides with the occurrence data in Brazil, and the highest RQs were found for insecticides, which agrees with the present work. 

## 4. Conclusions

Of the 27 pesticides in the Brazilian directive for the classification of surface freshwater (CONAMA 357/2005), 17 have RQs higher than the LOC for at least one of the tested organisms, indicating that the MVs are not safe for the biota. Many of these pesticides, including the persistent organochlorines, have been banned in Brazil and are considered POPs; however, the herbicides alachlor, atrazine, 2,4-D and glyphosate, as well as the insecticides carbaryl and malathion, are still authorized in the country. Thus, it is necessary to review the MVs established in the legislation, so that the objectives for the uses of water classes 1/2 and 3 are preserved, in addition to the protection of aquatic ecosystems. 

Furthermore, in addition to the 12 POPs, seven pesticides included in the Brazilian directive are no longer registered in the country. This indicates a necessary revision of the legislation, taking into account the pesticides that are currently most used and most found in water bodies in the country. In this context, this study may guide similar work in other countries and can help in the management of standard directives related to the uses of surface freshwaters, as well as in managing the protection and/or maintenance of aquatic ecosystems.

## Figures and Tables

**Table 1:** Pesticides included in CONAMA standard directive 357/05 for surface freshwater: registration situation in Brazil or persistent organic pollutant (POP) and maximum value concentrations according to the water use classification.

Pesticide ^a^	Current Situation ^b,c^	Maximum Value, µg/L ^a^	
		Class 1/2	Class 3
Alachlor	Registered: Environmental class II ^c^	20	-
Atrazine	Registered: Environmental class I–III ^c^	2	2
Carbaryl	Registered: Environmental class II ^c^	0.02	70
2,4-D	Registered: Environmental class I–III ^c^	4	30
Glyphosate	Registered: Environmental class I–III ^c^	65	280
Malathion	Registered: Environmental class I–IV ^c^	0.1	100
Simazine	Registered: Environmental class II–III ^c^	2	-
Trifuraline	Registered: Environmental class I–II ^c^	0.2	-
2,4,5-TP (fenoprop)	Not registered	10	10
Metolachlor	Not registered	10	-
Methoxychlor	Not registered	0.03	20
Demeton (demeton-O, demeton-S)	Not registered	0.1	14
Gution (azinphos methyl)	Not registered	0.005	0.005
Parathion	Not registered	0.04	35
2,4,5–T	Not registered	2	2
Aldrin	POP	0.005	0.03
Chlordane (cis, trans)	POP	0.04	0.3
DDT (p,p’-DDT, p,p’-DDE, p,p’-DDD)	POP	0.002	1
2,4-Dichlorophenol	POP	0.3	-
Dieldrin	POP	0.005	0.03
Endosulfan (I, II, sulphate)	POP	0.056	0.22
Endrin	POP	0.004	0.2
Heptachlor +heptachlor epoxide	POP	0.000039/0.01	0.03
Hexachlorobenzene	POP	0.00029/0.0065	-
Lindane (γ-HCH)	POP	0.02	2
Pentachlorophenol	POP	3/9	9
Toxaphene	POP	0.00028/0.01	0.21

^a^ Brazil [8]; ^b^ ANVISA [24] and ^c^ MAPA [19]; Environmental classification—I: extremely hazardous, II: highly hazardous, III; moderately hazardous; IV: slightly hazardous [19]; POP=persistent organic pollutant, United Nations Stockholm Convention (http://chm.pops.int/TheConvention/ThePOPs/ListingofPOPs/tabid/2509/Default.aspx (accessed on 24 November 2022)).

## Data Availability

Not applicable.

## Supplementary Materials

The following supporting information can be downloaded at: https://www.mdpi.com/article/10.3390/toxics10120767/s1, Table S1: Brazilian surface freshwater classes and their respective uses, in accordance with CONAMA standard directive 357/2005 [8]. Table S2: Pesticides listed in CONAMA directive 357/05, for which the risk quotient is higher than the level of concern (LOC) for at least one tested organism. LOC = 0.5 for acute risk of aquatic animals; LOC = 1 for chronic risk of aquatic animals and 1 for acute risk of plants [7]
