# Correction: de Araújo et al. Relationship between Pesticide Standards for Classification of Water Bodies and Ecotoxicity: A Case Study of the Brazilian Directive. *Toxics* 2022, *10*, 767

**DOI:** 10.3390/toxics11070601

**Published:** 2023-07-11

**Authors:** Esmeralda Pereira de Araújo, Eloisa Dutra Caldas, Eduardo Cyrino Oliveira-Filho

**Affiliations:** 1 Faculty of Planaltina—FUP, University of Brasilia—UnB, Federal District, Planaltina 73345-010, Brazil; esmeraldaneta.a@gmail.com; 2 Toxicology Laboratory, Faculty of Health Sciences, University of Brasília—UnB, Federal District, Brasília 70910-900, Brazil; 3 Brazilian Agricultural Research Corporation—Embrapa Cerrados, Federal District, Planaltina 73310-970, Brazil

**1.**  **Error in Table**

In the original publication, there was a mistake in “Table 2” as published. The endpoint value for glyphosate is not 12 µg/L, but 12,000 µg/L, hence glyphosate does not represent a risk to the biota and should not be include in the Table. The corrected Table 2 appears below.

**2.**  **Additional Affiliation(s)**

In the published publication, there was an error regarding the affiliation for Eduardo Cyrino Oliveira-Filho. The updated affiliation(s) should correct “ReseArch” to “Research”.

**3.**  **Text Correction**

There was an error in the original publication: “The results indicated that of the 27 pesticides included in the standard directive, 17 have a risk quotient (RQ) higher than the level of concern for at least one ecotoxicological parameter and may not protect the aquatic biota”.

A correction has been made to Abstract, Line 11: “The results indicated that of the 27 pesticides included in the standard directive, 16 have a risk quotient (RQ) higher than the level of concern for at least one ecotoxicological parameter and may not protect the aquatic biota.”

There was an error in the original publication: “Table 2 shows the 17 pesticides that have a RQ higher than the LOC for at least one organism tested, indicating that the biota may not be protected when present in an aquatic environment with concentrations at the legal levels.”

A correction has been made to 3. Results and Discussion, Paragraph 5: “Table 2 shows the 16 pesticides that have an RQ higher than the LOC for at least one organism tested, indicating that the biota may not be protected when present in an aquatic environment with concentrations at the legal levels.”

There was an error in the original publication: “Of the 27 pesticides in the Brazilian directive for the classification of surface freshwater (CONAMA 357/2005), 17 have RQs higher than the LOC for at least one of the tested organisms, indicating that the MVs are not safe for the biota.”

A correction has been made to 4. Conclusions, Paragraph 1: “Of the 27 pesticides in the Brazilian directive for the classification of surface freshwater (CONAMA 357/2005), 16 have RQs higher than the LOC for at least one of the tested organisms, indicating that the MVs are not safe for the biota.”

There was an error in the original publication: “In Brazil, CONAMA directive No. 357, from 17 MArch. 2005, determines the quality parameters, including the establishment of maximum values (MV) for pesticides in surface freshwater classes 1/2 and 3, which are destined for multiple uses (Table S1).”

A correction has been made to 1. Introduction, Paragraph 2: “In Brazil, CONAMA directive No. 357, from 17 March 2005, determined quality parameters, including the establishment of maximum values (MV) for pesticides in surface freshwater classes 1/2 and 3, which are destined for multiple uses (Table S1).”

There was an error in the original publication: “In order to carry out this study, reseArch. was done in the Web of Science, Scopus and Google Scholar databases, using the descriptors “aquatic toxicity”, “chronic” “acute”, “LC50”, “EC50”, “NOEL”, “NOEC” and the name of each pesticide listed in Table 1.”

A correction has been made to 2. Materials and Methods, Paragraph 1: “In order to carry out this study, research was conducted in the Web of Science, Scopus and Google Scholar databases, using the descriptors “aquatic toxicity”, “chronic” “acute”, “LC50”, “EC50”, “NOEL”, “NOEC” and the name of each pesticide listed in Table 1.”

There was an error in the original publication: “Considering the trophic levels, the group of secondary consumers is the one that shows a RQ higher than 1 (Table 2).”

A correction has been made to 3. Results and Discussion, Paragraph 6: “Considering the trophic levels, the group of primary consumers is the one that shows an RQ higher than 1 (Table 2).”

The authors state that the scientific conclusions are unaffected. These corrections were approved by the Academic Editor. The original publication [1] has also been updated.

## Figures and Tables

**Table 2:** Pesticides listed in CONAMA directive 357/05, for which the risk quotient is higher than the level of concern (LOC) for at least one tested organism. LOC = 0.5 for acute risk to aquatic animals; LOC = 1 for chronic risk to aquatic animals and 1 for acute risk to plants [7].

Pesticide	Risk Quotient Class 1,2/3 (µg/L)	Endpoint: Concentration (µg/L)	Tested Organism	Reference
Alachlor	3/-	EC_50_ (72 h): 6.69	*Raphidocelis subcapitata* ^a^	[26]
	2/-	EC_50_ (96 h): 10	*Raphidocelis subcapitata* ^a^	[27]
	2/-	EC_50_ (7 d)-biomass: 10	*Lemna minor* ^a^	[20]
	12.2/-	EC_50_ (<10 d): 1.64	Nonvascular plants ^a^	[7]
	8.7/-	EC_50_ (<10 d): 2.3	Vascular plants ^a^	[7]
Aldrin	-/3	NOEC-ratio of ovigerous to non-ovigerous females: 0.01	*Brachionus calyciflorus* ^b^	[31]
	-/1.8	LC_50_ (96 h): 0.017	*Pimephales promelas* ^c^	[21]
Dieldrin	5/30	LOEC-population growth rate: 0.001	*Brachionus calyciflorus* ^b^	[31]
	5/30	NOEC-ratio of ovigerous to non-ovigerous females: 0.001	*Brachionus calyciflorus* ^b^	[31]
	-/3	LOEC-ratio of ovigerous to non-ovigerous females: 0.01	*Brachionus calyciflorus* ^b^	[31]
Atrazine	2/2	EC_50_ (<10 d): <1	Nonvascular plants ^a^	[7]
Carbaryl	-/1.2	NOEC-resting egg production: 60	*Brachionus calyciflorus* ^b^	[37]
	-/3.5	NOEC-resting egg hatching rate: 20	*Brachionus calyciflorus* ^b^	[37]
	-/1.2	LOEC-resting egg hatching rate: 60	*Brachionus calyciflorus* ^b^	[37]
	-/41.2	EC_50_ or LC_50_ (48 or 96 h): 1.7	Invertebrates ^b^	[7]
	-/140	NOAEC: 0.5	Invertebrates ^b^	[7]
	-/10.9	EC_50_ (48 h): 6.4	*Daphnia pulex* ^b^	[20]
	-/12.3	LC_50_ (96 h): 5.7	*Americamysis bahia* ^b^	[20]
	-/11.7	NOAEC: 6	Fish ^c^	[7]
Chlordane	-/2.4	LC_50_ (96 h): 0.127	*Neocaridina denticulate* ^b^	[43]
	-/1.7	NOEC (14 d)-survival: 0.18	*Ceriodaphnia dubia* ^b^	[44]
	-/1.7	NOEC (14 d)- number of offspring per female: 0.18	*Ceriodaphnia dubia* ^b^	[44]
	-/1.7	NOEC (21 d)- number of offspring per female: 0.18	*Daphnia magna* ^b^	[44]
	-/4.3	LC_50_ (48 h)-trans: 0.07	*Daphnia* ^b^	[21]
	-/7.5	LC_50_ (96 h)-trans: 0.04	*Pimephales promelas* ^c^	[21]
2,4-D	-/1	LOEC: 29	*Hyalella meinerti* ^b^	[48]
	-/1	NOEC: <29	*Hyalella meinerti* ^b^	[48]
	1.2/9.3	LC_50_ (48 h): 3.22	*Daphnia* ^b^	[21]
	1.5/11.6	LC_50_ (96 h): 2.59	*Pimephales promelas* ^c^	[21]
Demeton	-/1.3	EC_50_ (48 h) ^d^: 10.4	*Daphnia pulex* ^b^	[20]
	-/1.6	LC_50_ (48 h) ^d1^: 8.62	*Daphnia* ^b^	[21]
	-/3.2	LC_50_ (96 h) ^d1^: 4.43	*Pimephales promelas* ^c^	[21]
	-/3.2	LC_50_ (48 h) ^d2^: 4.44	*Daphnia* ^b^	[21]
DDT	-/1	EC_50_ (48 h) ^e^: 1	*Bosmina longirostris* ^b^	[20]
Endosulfan	5.6/22	NOAEC: 0.01	Invertebrates ^b^	[7]
	0.6/2.2	LC_50_ (96 h): 0.1	Fish ^c^	[7]
	2.4/9.6	NOAEC: 0.023	Fish ^c^	[7]
	155.6/611.1	LC_50_ (96 h): 0.00036	*Chironomus ramosus ^b^*	[52]
	112/440	NOEC (28 d): 0.0005	*Cyprinodon variegatus* ^c^	[20]
Endrin	-/1.1	LC_50_ (48 h): 0.19	*Daphnia* ^b^	[21]
	2/100	LC_50_ (96 h): 0.002	*Pimephales promelas* ^c^	[21]
	-/1.7	NOEC (21 d): 0.12	*Cyprinodon variegatus* ^c^	[20]
Lindane	-/2	EC_50_ or LC_50_ (48 or 96 h): 1	*Invertebrates* ^b^	[7]
	-/1.2	LC_50_ (96 h): 1.7	Fish ^c^	[7]
	-/0.7	LC_50_ (96 h): 2.9	*Oncorhynchus mykiss* ^c^	[20]
Malathion	1/1020.4	EC_50_ or LC_50_ (48 or 96 h): 0.098	Invertebrates ^b^	[7]
	1.7/1666.7	NOAEC: 0.06	Invertebrates ^b^	[7]
	-/111.1	LC_50_ (48 h): 0.9	*Daphnia magna* ^b^	[57]
	-/4.9	LC_50_ (48 h): 20.32	*Daphnia* ^b^	[21]
	-/142.9	EC_50_ (48 h): 0.7	*Daphnia magna* ^b^	[20]
	1.7/1666.7	NOEC (21 d): 0.06	*Daphnia magna* ^b^	[20]
	-/66.7	LC_50_ (96 h): 1.5	*Americamysis bahia* ^b^	[20]
	-/24.4	LC_50_ (96 h): 4.1	Fish ^c^	[7]
	-/11.6	NOAEC: 8.6	Fish ^c^	[7]
	3125/3,125,000	LC_50_ (96 h): 0.000032	*Chironomus ramosus* ^b^	[52]
	-/22.3	LC_50_ (96 h): 4.48	*Pimephales promelas* ^c^	[21]
	-/5.6	LC_50_ (96 h): 18	*Oncorhynchus mykiss* ^c^	[20]
	-/1.1	NOEC (21 d): 91	*Oncorhynchus mykiss* ^c^	[20]
Metolachlor	1.3/-	EC_50_ (<10 d): 8	Nonvascular Plants ^a^	[7]
	10/-	NOAEC: 1	Invertebrates ^b^	[7]
Metoxichlor	-/0.7	LC_50_ (48 h): 30	*Daphnia* ^b^	[21]
	-/14.3	EC_50_ or LC_50_ (48 or 96 h): 1.4	Invertebrates ^b^	[7]
	-/25.6	EC_50_ (48 h): 0.78	*Daphnia magna* ^b^	[20]
	-/20	NOEC (21 d): 1	*Daphnia magna* ^b^	[20]
	-/1.3	LC_50_ (96 h): 15	Fish ^c^	[7]
Parathion	-/92.1	LC_50_ (48 h): 0.38	*Daphnia magna* ^b^	[57]
	-/46.7	LC_50_ (48 h): 0.75	*Daphnia* ^b^	[21]
	-/14	EC_50_ (48 h): 2.5	*Daphnia magna* ^b^	[20]
	-/350	NOEC (21 d): 0.1	*Daphnia magna* ^b^	[20]
	-/318.2	LC_50_ (96 h): 0.11	*Americamysis bahia* ^b^	[20]

d: day; h: hour; LC_50_: lethal concentration; EC_50_: effective concentration; LOEC: lowest observed effect concentration; NOAEC: no observed adverse effect concentration; NOEC: no observed effect concentration; LOEC: lowest observed effect concentrations. ^a^ Producer organism; ^b^ Primary consumer; ^c^ Secondary consumer; ^d^ Demeton; ^d1^ Isomer S; ^d2^ Isomer O; ^e^ Degradation product of DDE. All ecotoxicological studies were conducted in a laboratory setting, except for Refs. [7,20,21], where this information was not available.
