# Supplementary material for: Relationship between Pesticide Standards for Classification of Water Bodies and Ecotoxicity: A Case Study of the Brazilian Directive
Source: Toxics. 2022 Dec 8;10(12):767. doi: 10.3390/toxics10120767 (PMC9783844; doi:10.3390/toxics10120767)
Supplement: Supplementary file 1 [file toxics-10-00767-s001.zip › toxics-2031504-supplementary.pdf]

Supplementary Materials: Relationship between Pesticide Standards for Classification of Water Bodies and Ecotoxicity: A Case Study of the Brazilian Directive

Esmeralda Pereira de Araújo, Eloisa Dutra Caldas and Eduardo Cyrino Oliveira-Filho

Table S1. Brazilian surface freshwater classes and their respective uses, in accordance with CONAMA standard directive 357/2005[1].

**Special class**	**Class 1**	**Class 2**	**Class 3**	**Class 4**
Human supply after disinfection	Human supply after simplified treatment	Human supply after conventional treatment	Human supply after advanced conventional treatment	Navigation
Preservation of the natural equilibrium of aquatic communities	Protection of aquatic communities	Protection of aquatic communities	Irrigation of arboreal, cereal and forage crops	Landscape harmony
Preservation of aquatic environments in completely protected conservation units	Primary contact recreation	Primary contact recreation	Amateur fishing	
	Irrigation of vegetables that are consumed raw and fruit that develops roots in the ground and is eaten raw without removing the skin	Irrigation of vegetables, fruiting plants and those in parks, gardens, sports grounds and leisure areas in which the public can have direct contact	Secondary contact recreation	
	Protection of aquatic communities in Indigenous Lands	Aquiculture and fishing	Provision of water for rearing animals	

Table S2: Ecotoxicology data for the pesticides listed in CONAMA standard directive 357/05 and the the risk quotient (RQ) is higher than the level of concern (LOC) for at least one tested organism. LOC = 0.5 for acute risk of aquatic animals; LOC = 1 for chronic risk of aquatic animals and 1 for acute risk for of plants [2].

Trophic level	Pesticide	RQ Class 1/2	RQ Class 3	Endpoint: concentration (µg/L)	Tested organism	Life stage	Reference
P	Alachlor	**2.99**	-	EC50 (72h): 6.69	*Raphidocelis subcapitata*	n.i.	[3]
P	Alachlor	**2**	-	EC50 (96h): 10	*Raphidocelis subcapitata*	n.i.	[4]
P	Alachlor	0.7692	-	EC50 (96h): 26	*Chlorella vulgaris*	n.i.	[4]
P	Alachlor	0.0434	-	EC50 (96h): 461	*Chlamydomonas reinhardi*	n.i.	[4]
P	Alachlor	0.0151	-	EC50 (96h): 1328	*Scenedesmus quadricauda*	n.i.	[4]
P	Alachlor	0.0067	-	EC50 (96h): >3000	*Microcystis sp.*	n.i.	[4]
P	Alachlor	0.0067	-	EC50 (96h): >3000	*Anabaena flosque*	n.i.	[4]
P	Alachlor	0.0415	-	EC50 (14d): 482	*Lemna minor*	n.i.	[4]
P	Alachlor	0.2353	-	EC50 (14d): 85	*Ceratophyllum demersum*	n.i.	[4]
P	Alachlor	0.0067	-	EC50 (14d): >3000	*Elodea canadensis*	n.i.	[4]
P	Alachlor	0.0067	-	EC50 (14d): >3000	*Myriophyllum heterophyllum*	n.i.	[4]
P	Alachlor	0.0342	-	EC50 (14d): 584	*Najas sp.*	n.i.	[4]
P	Alachlor	**2**	-	EC50 (7d)-biomass: 10	*Lemna minor*	n.i.	[5]
P	Alachlor	0.0207	-	EC50 (72h)-growth: 966	*Scenedesmus quadricauda*	n.i.	[5]
P	Alachlor	**1**	-	NOEC (96h)-growth: 20	*Chlorella pyrenoidosa*	n.i.	[5]
P	Alachlor	**12.20**	-	EC50 (<10d): 1.64	Nonvascular Plants	n.i.	[6]
P	Alachlor	0.0070	-	EC50 (72h): 2868.12	Algae	n.i.	[7]
PC	Alachlor	0.0029	-	LC50 (48h): 6900.92	*Daphnia*	n.i.	[7]
P	Alachlor	**8.70**	-	EC50 (<10d): 2.3	Vascular plants	n.i.	[6]
PC	Alachlor	0.0080	-	EC50 or LC50 (48 or 96h): 2500	Invertebrates	n.i.	[6]
PC	Alachlor	0.1818	-	NOAEC: 110	Invertebrates	n.i.	[6]
PC	Alachlor	0.0027	-	EC50 (48h): 7500	*Daphnia magna*	Neonate	[3]
PC	Alachlor	0.0042	-	EC50 (48h): 4790	*Hyalella azteca*	Juvenile	[8]
PC	Alachlor	0.0032	-	EC50 (48h): 6300	*Ceriodaphnia dubia*	Neonate	[8]
PC	Alachlor	0.0010	-	EC50 (48h): 21000	*Daphnia magna*	Neonate	[8]
PC	Alachlor	0.0027	-	EC50 (96h): 7400	*Physa gyrina*	Juvenile	[8]

Trophic level	Pesticide	RQ Class 1/2	RQ Class 3	Endpoint: concentration (µg/L)	Tested organism	Life stage	Reference
PC	Alachlor	0.0021	-	EC50 (96h): 9370	*Lymnaea stagnalis*	Juvenile	[8]
PC	Alachlor	0.0019	-	EC50 (24h): 10300	*Thamnocephalus platyurus*	Nauplii or early-instar juvenile	[8]
SC	Alachlor	0.0017	-	LC50 (96h): 11500	*Rana pipiens*	Larvae-early	[9]
SC	Alachlor	0.0057	-	LC50 (96h): 3500	*Rana pipiens*	Larvae-late	[9]
SC	Alachlor	0.0051	-	LC50 (96h): 3900	*Bufo americanus*	Larvae-early	[9]
SC	Alachlor	0.0061	-	LC50 (96h): 3300	*Bufo americanus*	Larvae-late	[9]
SC	Alachlor	0.0022	-	LC50 (96h): 9100	*Oncorhynchus mykiss*	n.i.	[9]
SC	Alachlor	0.0012	-	LC50 (96h): 16700	*Ictalurus punctatus*	n.i.	[9]
SC	Alachlor	0.0100	-	PNOEC (30d): 2000	*Rana pipiens*	Larvae-early	[9]
SC	Alachlor	0.0426	-	PNOEC (30d): 470	*Rana pipiens*	Larvae-late	[9]
SC	Alachlor	0.0143	-	PNOEC (30d): 1400	*Bufo americanus*	Larvae-early	[9]
SC	Alachlor	0.0426	-	PNOEC (30d): 470	*Bufo americanus*	Larvae-late	[9]
PC	Alachlor	0.0020	-	EC50 (48h): 10000	*Daphnia magna*	n.i.	[5]
SC	Alachlor	0.0111	-	LC50 (96h): 1800	Fish	n.i.	[6]
SC	Alachlor	0.1070	-	NOAEC: 187	Fish	n.i.	[6]
SC	Alachlor	0.0524	-	LC50 (96h): 381.9	*Oreochromis niloticus*	2.5 months old	[10]
SC	Alachlor	0.0571	-	MATC: 350	*Oreochromis niloticus*	2.5 months old	[10]
SC	Alachlor	0.0143	-	PNOEC (30d): 1400	*Oncorhynchus mykiss*	n.i.	[9]
SC	Alachlor	0.0211	-	PNOEC (30d): 950	*Ictalurus punctatus*	n.i.	[9]
SC	Alachlor	0.0111	-	LC50 (96h): 1800	*Oncorhynchus mykiss*	n.i.	[5]
SC	Alachlor	0.0043	-	LC50 (96h): 4607.91	*Pimephales promelas*	n.i.	[7]
P	Aldrin	0.0050	0.0300	NOEC (24h)- population growth rate: 1	*Brachionus calyciflorus*	Adult	[11]
P	Aldrin	0.0005	0.0030	LOEC-population growth rate: 10	*Brachionus calyciflorus*	Adult	[11]
P	Aldrin	0.5000	**3**	NOEC-ratio of ovigerous females to non-ovigerous females: 0.01	*Brachionus calyciflorus*	Adult	[11]

Trophic level	Pesticide	RQ Class 1/2	RQ Class 3	Endpoint: concentration (μg/L)	Tested organism	Life stage	Reference
P	Aldrin	0.0500	0.3000	LOEC-ratio of ovigerous females to non-ovigerous females: 0.1	*Brachionus calyciflorus*	Adult	[11]
P	Aldrin	0.0002	0.0010	EC50: 31.12	*Brachionus calyciflorus*	Adult	[11]
P	Aldrin	0.0115	0.0688	EC50 (72h): 0.436	Algae	n.i.	[7]
PC	Aldrin	0.0521	0.3125	LC50 (48h): 0.096	*Daphnia*	n.i.	[7]
PC	Aldrin	0.0002	0.0011	EC50 (48h): 28	*Daphnia magna*	n.i.	[5]
SC	Aldrin	0.0500	0.3000	NOEC-growth: 0.1	*Danio rerio*	Juvenile	[12]
SC	Aldrin	0.0050	0.0300	OEC-growth: 1	*Danio rerio*	Juvenile	[12]
SC	Aldrin	0.0003	0.0016	LC50 (96h): 18.5	*Cyprinus carpio*	n.i.	[13]
SC	Aldrin	0.0002	0.0011	LC50 (96h): 27	*Puntius ticto*	n.i.	[13]
SC	Aldrin	0.2941	**1.76**	LC50 (96h): 0.017	*Pimephales promelas*	n.i.	[7]
SC	Aldrin	0.0011	0.0065	LC50 (96h): 4.6	*Lepomis macrochirus*	n.i.	[5]
P	Dieldrin	0	0	NOEC-population growth rate: 0	*Brachionus calyciflorus*	Adult	[11]
P	Dieldrin	**5.00**	**30**	LOEC-population growth rate: 0.001	*Brachionus calyciflorus*	Adult	[11]
P	Dieldrin	**5.00**	**30**	NOEC-ratio of ovigerous females to non-ovigerous females: 0.001	*Brachionus calyciflorus*	Adult	[11]
P	Dieldrin	0.5000	**3**	LOEC-ratio of ovigerous females to non-ovigerous females: 0.01	*Brachionus calyciflorus*	Adult	[11]
P	Dieldrin	0.00001	0.00003	EC50: >>1000	*Brachionus calyciflorus*	Adult	[11]
P	Dieldrin	0.0077	0.0462	EC50 (72h): 0.65	Algae	n.i.	[7]
P	Dieldrin	0.0001	0.0003	EC50 (72h)-growth: 100	*Chlorella pyrenoidosa*	n.i.	[5]
PC	Dieldrin	0.0263	0.1579	LC50 (48h): 0.19	*Daphnia*	n.i.	[7]
PC	Dieldrin	0.00002	0.0001	LC50 (48h): 250	*Daphnia magna*	n.i.	[5]
PC	Dieldrin	0.0008	0.0050	LC50 (24h): >6	*Hyalella azteca*	Adult	[14]
SC	Dieldrin	0.0003	0.0020	LC50 (21d): 14.9	*Xenopus laevis*	Embryo-larval	[15]
SC	Dieldrin	0.0001	0.0007	LC50 (4d): 40.4	*Xenopus laevis*	Tadpole	[15]
SC	Dieldrin	0.0001	0.0009	LC50 (21d): 34.4	*Rana catesbeiana*	Embryo-larval	[15]
SC	Dieldrin	0.0006	0.0034	LC50 (4d): 8.7	*Rana catesbeiana*	Tadpole	[15]

Trophic level	Pesticide	RQ Class 1/2	RQ Class 3	Endpoint: concentration (µg/L)	Tested organism	Life stage	Reference
SC	Dieldrin	0.0001	0.0004	LC50 (4d): 71.3	*Rana pipiens*	Tadpole	[15]
SC	Dieldrin	0.0008	0.0050	NOAEL (4d): 6	*Xenopus laevis*	Embryo larval-length	[15]
SC	Dieldrin	0.0002	0.0012	LOAEL (4d): 24.1	*Xenopus laevis*	Embryo larval-length	[15]
SC	Dieldrin	0.0002	0.0009	NOAEL (4d): 31.6	*Xenopus laevis*	Tadpole-mortality	[15]
SC	Dieldrin	0.0001	0.0004	LOAEL (4d): 82.9	*Xenopus laevis*	Tadpole-mortality	[15]
SC	Dieldrin	0.0002	0.0012	LOAEL (4d): 25.4	*Rana catesbeiana*	Embryo larval-mortality	[15]
SC	Dieldrin	0.0005	0.0027	NOAEL (4d): 11	*Rana catesbeiana*	Embryo larval-mortality	[15]
SC	Dieldrin	0.0004	0.0027	LOAEL (4d): 11.2	*Rana catesbeiana*	Tadpole-mortality	[15]
SC	Dieldrin	0.0013	0.0075	NOAEL (4d): 4	*Rana catesbeiana*	Tadpole-mortality	[15]
SC	Dieldrin	0.0042	0.0250	LOAEL (10d): 1.2	*Xenopus laevis*	Tadpole chronic-mortality	[15]
SC	Dieldrin	0.0063	0.0375	NOAEL (10d): 0.8	*Xenopus laevis*	Tadpole chronic-mortality	[15]
SC	Dieldrin	0.0012	0.0073	LOAEL (28d): 4.1	*Rana pipiens*	Tadpole chronic-mortality	[15]
SC	Dieldrin	0.0026	0.0158	NOAEL (28d): 1.9	*Rana pipiens*	Tadpole chronic-mortality	[15]
SC	Dieldrin	0.0005	0.0031	LC50 (96h): 9.7	*Cyprinus carpio*	n.i.	[13]
SC	Dieldrin	0.0003	0.0017	LC50 (96h): 18	*Puntius ticto*	n.i.	[13]
SC	Dieldrin	0.0042	0.0250	LC50 (96h): 1.2	*Oncorhynchus mykiss*	n.i.	[5]

Trophic level	Pesticide	RQ Class 1/2	RQ Class 3	Endpoint: concentration (μg/L)	Tested organism	Life stage	Reference
P	Atrazine	0.0004	0.0004	LC50 (7d): 5270	*Lemna minor*	n.i.	[16]
P	Atrazine	**2**	**2**	EC50 (<10d): <1	Nonvascular Plants	n.i.	[6]
P	Atrazine	0.4348	0.4348	EC50 (<10d): 4.6	Vascular plants	n.i.	[6]
P	Atrazine	0.00002	0.00002	LC50 (7d): >100000	*Azolla caroliniana*	n.i.	[16]
P	Atrazine	0.0171	0.0171	EC50 (96h): 117	*Raphidocelis subcapitata*	n.i.	[4]
P	Atrazine	0.0213	0.0213	EC50 (96h): 94	*Chlorella vulgaris*	n.i.	[4]
P	Atrazine	0.0114	0.0114	EC50 (96h): 176	*Chlamydomonas reinhardi*	n.i.	[4]
P	Atrazine	0.0118	0.0118	EC50 (96h): 169	*Scenedesmus quadricauda*	n.i.	[4]
P	Atrazine	0.0222	0.0222	EC50 (96h): 90	*Microcystis sp.*	n.i.	[4]
P	Atrazine	0.0007	0.0007	EC50 (96h): 3000	*Anabaena flosque*	n.i.	[4]
P	Atrazine	0.0217	0.0217	EC50 (14d): 92	*Lemna minor*	n.i.	[4]
P	Atrazine	0.0909	0.0909	EC50 (14d): 22	*Ceratophyllum demersum*	n.i.	[4]
P	Atrazine	0.0952	0.0952	EC50 (14d): 21	*Elodea canadensis*	n.i.	[4]
P	Atrazine	0.0152	0.0152	EC50 (14d): 132	*Myriophyllum heterophyllum*	n.i.	[4]
P	Atrazine	0.0833	0.0833	EC50 (14d): 24	*Najas sp.*	n.i.	[4]
P	Atrazine	0.0001	0.0001	LC50 (24h): 39200	*Brachionus calyciflorus*	Adult	[17]
P	Atrazine	0.0063	0.0063	NOEC-population growth rate: 320	*Brachionus calyciflorus*	Adult	[17]
P	Atrazine	0.0016	0.0016	LOEC-population growth rate: 1280	*Brachionus calyciflorus*	Adult	[17]
P	Atrazine	0.0250	0.0250	NOEC-resting egg hatching rate: 80	*Brachionus calyciflorus*	Adult	[17]
P	Atrazine	0.0063	0.0063	LOEC-resting egg hatching rate: 320	*Brachionus calyciflorus*	Adult	[17]
P	Atrazine	0.0250	0.0250	NOEC-resting egg production: 80	*Brachionus calyciflorus*	Adult	[17]
P	Atrazine	0.0063	0.0063	LOEC-resting egg production: 320	*Brachionus calyciflorus*	Adult	[17]
P	Atrazine	0.1361	0.1361	LC50 (96h): 14.7	*Scenedesmus obliquus*	n.i.	[18]
P	Atrazine	0.0294	0.0294	EC50 (72h): 68.02	Algae	n.i.	[7]
P	Atrazine	0.0339	0.0339	EC50 (72h)-growth: 59	*Raphidocelis subcapitata*	n.i.	[5]
P	Atrazine	0.1053	0.1053	EC50 (7d)-biomass: 19	*Lemna gibba*	n.i.	[5]
P	Atrazine	0.0200	0.0200	NOEC (96h)-growth: 100	Green algae	n.i.	[5]

Trophic level	Pesticide	RQ Class 1/2	RQ Class 3	Endpoint: concentration (μg/L)	Tested organism	Life stage	Reference
PC	Atrazine	0.0028	0.0028	EC50 or LC50 (48 or 96h): 720	Invertebrates	n.i.	[6]
PC	Atrazine	0.0333	0.0333	NOAEC: 60	Invertebrates	n.i.	[6]
PC	Atrazine	0.0002	0.0002	LC50 (96h): 9900	*Paratya australiensis*	n.i.	[19]
PC	Atrazine	0.00003	0.00003	LC50 (48h): 60600	*Daphnia carinata*	n.i.	[18]
SC	Atrazine	0.00004	0.00004	LC50 (96h): 47600	*Rana pipiens*	Larvae-early	[9]
SC	Atrazine	0.0001	0.0001	LC50 (96h): 14500	*Rana pipiens*	Larvae-late	[9]
SC	Atrazine	0.0001	0.0001	LC50 (96h): 26500	*Bufo americanus*	Larvae-early	[9]
SC	Atrazine	0.0002	0.0002	LC50 (96h): 10700	*Bufo americanus*	Larvae-late	[9]
SC	Atrazine	0.0001	0.0001	LC50 (96h): 20500	*Oncorhynchus mykiss*	n.i.	[9]
SC	Atrazine	0.0001	0.0001	LC50 (96h): 23800	*Ictalurus punctatus*	n.i.	[9]
SC	Atrazine	0.0004	0.0004	PNOEC (30d): 5100	*Rana pipiens*	Larvae-early	[9]
SC	Atrazine	0.0031	0.0031	PNOEC (30d): 650	*Rana pipiens*	Larvae-late	[9]
SC	Atrazine	0.0011	0.0011	PNOEC (30d): 1900	*Bufo americanus*	Larvae-early	[9]
SC	Atrazine	0.0029	0.0029	PNOEC (30d): 690	*Bufo americanus*	Larvae-late	[9]
PC	Atrazine	0.00003	0.00003	LC50 (96h): 77900	*Pacifastacus leniusculus*	Juvenile	[20]
PC	Atrazine	0.0001	0.0001	LC50 (48h): 30031.17	*Daphnia*	n.i.	[7]
PC	Atrazine	0.00002	0.00002	LC50 (48h): 85000	*Daphnia magna*	n.i.	[5]
PC	Atrazine	0.0080	0.0080	LOEC (21d): 250	*Daphnia magna*	n.i.	[5]
PC	Atrazine	0.0020	0.0020	LC50 (96h): 1000	*Americamysis bahia*	n.i.	[5]
SC	Atrazine	0.0004	0.0004	LC50 (96h): 5300	Fish	n.i.	[6]
SC	Atrazine	0.4000	0.4000	NOAEC: 5	Fish	n.i.	[6]
SC	Atrazine	0.0010	0.0010	PNOEC (30d): 2000	*Oncorhynchus mykiss*	n.i.	[9]
SC	Atrazine	0.0005	0.0005	PNOEC (30d): 4300	*Ictalurus punctatus*	n.i.	[9]
SC	Atrazine	0.0002	0.0002	LC50 (96h): 9620	*Silurana tropicalis*	Tadpole	[21]
SC	Atrazine	0.0002	0.0002	LC50 (96h): 10200	*Rhamdia quelen*	Fingerling	[22]
SC	Atrazine	0.0001	0.0001	LC50 (96h): 13918.28	*Pimephales promelas*	n.i.	[7]
SC	Atrazine	0.0004	0.0004	LC50 (96h): 4500	*Oncorhynchus mykiss*	n.i.	[5]

Trophic level	Pesticide	RQ Class 1/2	RQ Class 3	Endpoint: concentration (µg/L)	Tested organism	Life stage	Reference
SC	Atrazine	0.0010	0.0010	NOEC (21d): 2000	*Oncorhynchus mykiss*	n.i.	[5]
P	Carbaryl	0.000005	0.0171	LC50 (24h): 4100	*Brachionus calyciflorus*	Adult	[17]
P	Carbaryl	0.00003	0.1061	EC50 (<10d): 660	Nonvascular Plants	n.i.	[6]
P	Carbaryl	0.0003	**1.17**	NOEC-resting egg production: 60	*Brachionus calyciflorus*	Adult	[17]
P	Carbaryl	0.0001	0.3889	LOEC-resting egg production: 180	*Brachionus calyciflorus*	Adult	[17]
P	Carbaryl	0.0010	**3.50**	NOEC-resting egg hatching rate: 20	*Brachionus calyciflorus*	Adult	[17]
P	Carbaryl	0.0003	**1.17**	LOEC-resting egg hatching rate: 60	*Brachionus calyciflorus*	Adult	[17]
P	Carbaryl	0.00001	0.0226	EC50 (72h): 3099.64	Algae	n.i.	[7]
P	Carbaryl	0.000001	0.0051	EC50 (7d)-biomass: 13700	*Lemna gibba*	n.i.	[5]
P	Carbaryl	0.00003	0.1167	EC50 (72h)-growth: 600	*Chlorella spp.*	n.i.	[5]
PC	Carbaryl	0.0118	**41.18**	EC50 or LC50 (48 or 96h): 1.7	Invertebrates	n.i.	[6]
PC	Carbaryl	0.0400	**140**	NOAEC: 0.5	Invertebrates	n.i.	[6]
PC	Carbaryl	0.000001	0.0025	LC50 (48h): 28475.68	*Daphnia*	n.i.	[7]
PC	Carbaryl	0.0031	**10.94**	EC50 (48h): 6.4	*Daphnia pulex*	n.i.	[5]
PC	Carbaryl	0.0001	0.2800	NOEC (21d): 250	*Daphnia magna*	n.i.	[5]
PC	Carbaryl	0.0035	**12.28**	LC50 (96h): 5.7	*Americamysis bahia*	n.i.	[5]
SC	Carbaryl	0.0001	0.3182	LC50 (96h): 220	Fish	n.i.	[6]
SC	Carbaryl	0.0033	**11.67**	NOAEC: 6	Fish	n.i.	[6]
SC	Carbaryl	0.000002	0.0076	LC50 (96h): 9233.63	*Pimephales promelas*	n.i.	[7]
SC	Carbaryl	0.00001	0.0269	LC50 (96h): 2600	*Pimephales promelas*	n.i.	[5]
SC	Carbaryl	0.0001	0.3333	NOEC (34d): 210	*Pimephales promelas*	n.i.	[5]
P	Chlordane (cis e trans)	0.0002	0.0013	EC50 (72h): 235.72	Algae	n.i.	[7]
P	Chlordane	0.0714	0.5357	EC50 (72h)-cis: 0.56	Algae	n.i.	[7]
P	Chlordane	0.0714	0.5357	EC50 (72h)-trans: 0.56	Algae	n.i.	[7]
PC	Chlordane	0.3150	**2.36**	LC50 (96h): 0.127	*Neocaridina denticulata*	Juvenile	[23]
PC	Chlordane	0.0138	0.1034	NOEC (7d)-longevity: 2.9	*Ceriodaphnia dubia*	Neonate and adult	[24]

Trophic level	Pesticide	RQ Class 1/2	RQ Class 3	Endpoint: concentration (µg/L)	Tested organism	Life stage	Reference
PC	Chlordane	0.0057	0.0429	LOEC (7d)-longevity: 7	*Ceriodaphnia dubia*	Neonate and adult	[24]
PC	Chlordane	0.0041	0.0309	EC50 (21d)-longevity: 9.72	*Daphnia magna*	Neonate and adult	[24]
PC	Chlordane	0.0138	0.1034	NOEC (21d)-longevity: 2.9	*Daphnia magna*	Neonate and adult	[24]
PC	Chlordane	0.0057	0.0429	LOEC (21d)-longevity: 7	*Daphnia magna*	Neonate and adult	[24]
PC	Chlordane	0.2222	**1.67**	NOEC (14d)-survival: 0.18	*Ceriodaphnia dubia*	Neonate and adult	[24]
PC	Chlordane	0.0323	0.2419	EC50 (14d)-survival: 1.24	*Ceriodaphnia dubia*	Neonate and adult	[24]
PC	Chlordane	0.0548	0.4110	LOEC (14d)-survival: 0.73	*Ceriodaphnia dubia*	Neonate and adult	[24]
PC	Chlordane	0.0142	0.1068	EC50 (21d)-survival: 2.81	*Daphnia magna*	Neonate and adult	[24]
PC	Chlordane	0.0220	0.1648	NOEC (21d)-survival: 1.82	*Daphnia magna*	Neonate and adult	[24]
PC	Chlordane	0.0138	0.1034	LOEC (21d)-survival: 2.9	*Daphnia magna*	Neonate and adult	[24]
PC	Chlordane	0.2222	**1.67**	NOEC (14d)- number of offspring per female: 0.18	*Ceriodaphnia dubia*	Adult	[24]
PC	Chlordane	0.0548	0.4110	LOEC (14d)- number of offspring per female: 0.73	*Ceriodaphnia dubia*	Adult	[24]
PC	Chlordane	0.0105	0.0789	EC50 (14d)-number of offspring per female: 3.8	*Ceriodaphnia dubia*	Adult	[24]
PC	Chlordane	0.2222	**1.67**	NOEC (21d)- number of offspring per female: 0.18	*Daphnia magna*	Adult	[24]
PC	Chlordane	0.0548	0.4110	LOEC (21d)- number of offspring per female: 0.73	*Daphnia magna*	Adult	[24]
PC	Chlordane	0.0260	0.1948	EC50 (21d)-number of offspring per female: 1.54	*Daphnia magna*	Adult	[24]

Trophic level	Pesticide	RQ Class 1/2	RQ Class 3	Endpoint: concentration (µg/L)	Tested organism	Life stage	Reference
PC	Chlordane	0.0030	0.0224	EC50 (48h): 13.4	*Daphnia magna*	Neonate and <24h old	[25]
PC	Chlordane	0.0548	0.4110	LOEC (21d)-reproduction. number of offspring per female and brood size and on body length: 0.73	*Daphnia magna*	Neonate and <24h old	[25]
PC	Chlordane	0.0040	0.0297	EC50 (21d)-population growth rate: 10.1	*Daphnia magna*	Neonate and <24h old	[25]
PC	Chlordane	0.0009	0.0069	LC50 (48h): 43.63	*Daphnia*	n.i.	[7]
PC	Chlordane	0.0059	0.0446	LC50 (48h)-cis: 6.73	*Daphnia*	n.i.	[7]
PC	Chlordane	**0.57**	**4.29**	LC50 (48h)-trans: 0.07	*Daphnia*	n.i.	[7]
PC	Chlordane	0.0001	0.0005	EC50 (48h): 590	*Daphnia magna*	n.i.	[5]
PC	Chlordane	0.0006	0.0043	LOEC (21d): 70	*Daphnia magna*	n.i.	[5]
SC	Chlordane	0.0010	0.0077	LC50 (96h): 39	*Cyprinus carpio*	n.i.	[13]
SC	Chlordane	0.0008	0.0058	LC50 (96h): 52	*Puntius ticto*	n.i.	[13]
SC	Chlordane	0.0011	0.0081	LC50 (96h): 37.23	*Pimephales promelas*	n.i.	[7]
SC	Chlordane	0.0057	0.0428	LC50 (96h)-cis: 7.0089	*Pimephales promelas*	n.i.	[7]
SC	Chlordane	**1.00**	**7.50**	LC50 (96h)-trans: 0.04	*Pimephales promelas*	n.i.	[7]
SC	Chlordane	0.0004	0.0033	LC50 (96h): 90	*Oncorhynchus mykiss*	n.i.	[5]
P	2.4-D	0.00001	0.00004	LC50 (7d): 708350	*Azolla caroliniana*	n.i.	[26]
P	2.4-D	0.0134	0.1003	EC50 (<10d): 299.2	Vascular plants	n.i.	[6]
P	2.4-D	0.0017	0.0127	EC50 (24h)-growth rate: >2360	*Lemna minor*	n.i.	[27]
P	2.4-D	0.0017	0.0127	NOEC-growth rate: >2360	*Lemna minor*	n.i.	[27]
P	2.4-D	0.0003	0.0024	EC50 (72h): 12400.38	Algae	n.i.	[7]
P	2.4-D	0.0002	0.0012	EC50 (72h): 24200	*Raphidocelis subcapitata*	n.i.	[5]
P	2.4-D	0.0015	0.0111	EC50 (7d): 2700	*Lemna gibba*	n.i.	[5]
P	2.4-D	0.00004	0.0003	NOEC (96h)-growth: 100000	*Chlorella vulgaris*	n.i.	[5]
PC	2.4-D	0.0002	0.0012	EC50 or LC50 (48 or 96h): 25000	Invertebrates	n.i.	[6]
PC	2.4-D	0.00004	0.0003	LC50 (96h): >100000	*Hyalella meinerti*	7-14 days	[28]

Trophic level	Pesticide	RQ Class 1/2	RQ Class 3	Endpoint: concentration (µg/L)	Tested organism	Life stage	Reference
PC	2.4-D	0.1379	**1.03**	LOEC: 29	*Hyalella meinerti*	7-14 days	[28]
PC	2.4-D	0.1379	**1.03**	NOEC: <29	*Hyalella meinerti*	7-14 days	[28]
PC	2.4-D	**1.24**	**9.32**	LC50 (48h): 3.22	*Daphnia*	n.i.	[7]
PC	2.4-D	0.00003	0.0002	EC50 (48h): 134200	*Daphnia magna*	n.i.	[5]
PC	2.4-D	0.0001	0.0006	NOEC (21d): 46200	*Daphnia magna*	n.i.	[5]
SC	2.4-D	0.00005	0.0004	LC50 (96h): 81000	*Heteropneustes fossilis*	n.i.	[29]
SC	2.4-D	0.00003	0.0002	LC50 (96h): 122000	*Clarias batrachus*	n.i.	[29]
SC	2.4-D	0.00004	0.0003	LC50 (96h): 107000	*Channa punctatus*	n.i.	[29]
SC	2.4-D	0.00001	0.0001	LC50 (48h): 302000	*Culex pipiens fatigans*	Larvae	[29]
SC	2.4-D	**1.54**	**11.58**	LC50 (96h): 2.59	*Pimephales promelas*	n.i.	[7]
SC	2.4-D	0.00004	0.0003	LC50 (96h): 100000	*Pimephales promelas*	n.i.	[5]
SC	2.4-D	0.0001	0.0011	NOEC(28d): 27200	*Oryzias latipes*	n.i.	[5]
PC	Demeton (Demeton O + S)	0.0096	**1.35**	EC50 (48h): 10.4	*Daphnia pulex*	n.i.	[5]
SC	Demeton	0.0001	0.0088	LC50(96h): 1600	*Oncorhynchus mykiss*	n.i.	[5]
P	Demeton-S	0.0000	0.0005	EC50 (72h): 26127.64	Algae	n.i.	[7]
PC	Demeton-S	0.0116	**1.62**	LC50 (48h): 8.62	*Daphnia*	n.i.	[7]
SC	Demeton-S	0.0226	**3.16**	LC50 (96h): 4.43	*Pimephales promelas*	n.i.	[7]
P	Demeton-O	0.00001	0.0021	EC50 (72h): 6740.82	Algae	n.i.	[7]
PC	Demeton-O	0.0225	**3.15**	LC50 (48h): 4.44	*Daphnia*	n.i.	[7]
SC	Demeton-O	0.00004	0.0053	LC50 (96h): 2635.9	*Pimephales promelas*	n.i.	[7]
P	2.4-Dichlorophenol	0.00004	-	EC50 (72h); 6797.1	Algae	n.i.	[7]
P	2.4-Dichlorophenol	0.0002	-	EC50(7d)-biomass: 1500	*Lemna gibba*	n.i.	[5]
P	2.4-Dichlorophenol	0.0001	-	EC50 (72h)-growth: 3440	*Raphidocelis subcapitata*	n.i.	[5]
PC	2.4-Dichlorophenol	0.0001	-	LC50 (48h): 2570.63	*Daphnia*	n.i.	[7]
PC	2.4-Dichlorophenol	0.0001	-	EC50 (48h): 2800	*Daphnia magna*	n.i.	[5]
SC	2.4-Dichlorophenol	0.0010	-	MATC-survival: 290	*Pimephales promelas*	Early-juvenile	[30]
SC	2.4-Dichlorophenol	0.00005	-	LC50 (192h): 6500	*Pimephales promelas*	30-35 days old	[31]

Trophic level	Pesticide	RQ Class 1/2	RQ Class 3	Endpoint: concentration (µg/L)	Tested organism	Life stage	Reference
SC	2.4-Dichlorophenol	0.00003	-	LC50 (96h): 9728.8	*Pimephales promelas*	n.i.	[7]
SC	2.4-Dichlorophenol	0.0001	-	LC50 (96h): 2630	*Oncorhynchus mykiss*	n.i.	[5]
SC	DDT (p.p′-DDT. p.p′-DDE. p.p′-DDD)	0.00002	0.0077	NOEC (21d): 130	*Oncorhynchus mykiss*	n.i.	[5]
PC	DDE	0.0020	**1.00**	EC50 (48h): 1	*Bosmina longirostris*	n.i.	[5]
SC	DDE	0.0001	0.0313	LC50 (96h): 32	*Oncorhynchus mykiss*	n.i.	[5]
PC	DDD	0.0002	0.1111	EC50 (48h): >9	*Daphnia magna*	n.i.	[5]
SC	DDD	0.00003	0.0143	LC50 (96h): >70	*Oncorhynchus mykiss*	n.i.	[5]
PC	DDT	0.0004	0.2000	EC50 (48h): >5	*Daphnia magna*	n.i.	[5]
SC	DDT	0.000001	0.0004	LC50 (96h): >2500	*Oncorhynchus mykiss*	n.i.	[5]
P	p.p'-DDT	0.00001	0.0072	EC50 (72h): 138.26	Algae	n.i.	[7]
PC	p.p'-DDT	0.0001	0.0485	LC50 (48h): 20.62	*Daphnia*	n.i.	[7]
SC	p.p'-DDT	0.0008	0.4167	LC50 (96h): 2.4	*Cyprinus carpio*	n.i.	[13]
SC	p.p'-DDT	0.00004	0.0204	LC50 (96h): 49	*Puntius ticto*	n.i.	[13]
SC	p.p'-DDT	0.0001	0.0556	LC50 (96h): 17.98	*Pimephales promelas*	n.i.	[7]
P	Endosulfan (α + β + sulphate)	0.0257	0.1009	EC50 (72h): 2.18	Algae	n.i.	[7]
P	Endosulfan	0.0001	0.0005	EC50 (<10d): 428	Nonvascular Plants	n.i.	[6]
P	Endosulfan	0.00003	0.0001	EC50 (72h)-growth: 2150	*Anabaena doliolum*	n.i.	[5]
PC	Endosulfan	0.0933	0.3667	EC50 or LC50 (48 or 96h): 0.6	Invertebrates	n.i.	[6]
PC	Endosulfan	**5.60**	**22.00**	NOAEC: 0.01	Invertebrates	n.i.	[6]
PC	Endosulfan	0.0667	0.2619	LC50 (48h): 0.84	*Daphnia*	n.i.	[7]
PC	Endosulfan	0.0002	0.0009	LC50 (96h): 240	*Americamysis bahia*	n.i.	[5]
PC	Endosulfan	0.0001	0.0005	EC50 (48h): 440	*Daphnia magna*	n.i.	[5]
SC	Endosulfan	**0.56**	**2.20**	LC50 (96h): 0.1	Fish	n.i.	[6]
SC	Endosulfan	**2.43**	**9.57**	NOAEC: 0.023	Fish	n.i.	[6]
PC	Endosulfan	**155.56**	**611**	LC50 (96h): 0.00036	*Chironomus ramosus*	Larvae	[32]
SC	Endosulfan	0.0824	0.3235	LC50 (96h): 0.68	*Pimephales promelas*	n.i.	[7]

Trophic level	Pesticide	RQ Class 1/2	RQ Class 3	Endpoint: concentration (μg/L)	Tested organism	Life stage	Reference
SC	Endosulfan	0.0280	0.1100	LC50 (96h): 2	*Oncorhynchus mykiss*	n.i.	[5]
SC	Endosulfan	**112.00**	**440**	NOEC (28d): 0.0005	*Cyprinodon variegatus*	n.i.	[5]
P	α-Endosulfan	0.00003	0.0001	EC50 (72h): 2203.64	Algae	n.i.	[7]
P	α-Endosulfan	0.00003	0.0001	EC50 (72h)-growth: 2150	*Anabaena doliolum*	n.i.	[5]
PC	α-Endosulfan	0.0001	0.0003	LC50 (48h): 697.53	*Daphnia*	n.i.	[7]
PC	α-Endosulfan	0.0001	0.0005	EC50 (48h): 440	*Daphnia magna*	n.i.	[5]
PC	α-Endosulfan	0.0002	0.0009	LC50 (96h): 240	*Americamysis bahia*	n.i.	[5]
SC	α-Endosulfan	0.0001	0.0002	LC50 (96h): 981.35	*Pimephales promelas*	n.i.	[7]
SC	α-Endosulfan	0.0280	0.1100	LC50 (96h): 2	*Oncorhynchus mykiss*	n.i.	[5]
P	Endosulfan sulfate	0.0255	0.1000	EC50 (72h): 2.2	Algae	n.i.	[7]
PC	Endosulfan sulfate	0.0001	0.0003	EC50 (48h): 760	*Daphnia magna*	n.i.	[5]
PC	Endosulfan sulfate	0.0361	0.1419	LC50 (48h): 1.55	*Daphnia*	n.i.	[7]
SC	Endosulfan sulfate	0.0246	0.0968	LC50 (96h): 2.273	*Gambusia affinis*	n.i.	[33]
SC	Endosulfan sulfate	0.0272	0.1069	LC50 (96h): 2.058	*Heterandria formosa*	n.i.	[33]
SC	Endosulfan sulfate	0.0160	0.0627	LC50 (96h): 3.506	*Poecilia latipinna*	n.i.	[33]
SC	Endosulfan sulfate	0.0184	0.0722	LC50 (96h): 3.047	*Pimephales promelas*	n.i.	[33]
SC	Endosulfan sulfate	0.0560	0.2200	LC50 (96h): 1	*Pimephales promelas*	n.i.	[7]
SC	Endosulfan sulfate	0.0056	0.0220	LC50 (48h): 10	Cyprinidae	n.i.	[5]
P	Endrin	0.0062	0.3077	EC50 (72h): 0.65	Algae	n.i.	[7]
PC	Endrin	0.0211	**1.05**	LC50 (48h): 0.19	*Daphnia*	n.i.	[7]
PC	Endrin	0.0010	0.0476	EC50 (48h): 4.2	*Daphnia magna*	n.i.	[5]
SC	Endrin	0.0003	0.0154	EC50 (24h): 13	*Rana sphenocephala*	Larvae	[34]
SC	Endrin	0.0004	0.0222	LC50 (96h): 9	*Rana sphenocephala*	Larvae	[34]
SC	Endrin	0.0001	0.0050	EC50 (24h): >40	*Rana catesbeiana*	Larvae	[34]
SC	Endrin	0.0020	0.1000	LC50 (96h): 2	*Rana catesbeiana*	Larvae	[34]
SC	Endrin	0.0003	0.0125	EC50 (24h): <16	*Rana sylvatica*	Larvae	[34]
SC	Endrin	0.0001	0.0059	LC50 (96h): 34	*Rana sylvatica*	Larvae	[34]

Trophic level	Pesticide	RQ Class 1/2	RQ Class 3	Endpoint: concentration (µg/L)	Tested organism	Life stage	Reference
SC	Endrin	0.0005	0.0250	EC50 (24h): 8	*Bufo americanus*	Larvae	[34]
SC	Endrin	0.0004	0.0200	LC50 (96h): 10	*Bufo americanus*	Larvae	[34]
SC	Endrin	0.0002	0.0087	EC50 (24h): 23	*Acris crepitans*	Larvae	[34]
SC	Endrin	0.0004	0.0200	LC50 (96h): 10	*Acris crepitans*	Larvae	[34]
SC	Endrin	0.0002	0.0111	EC50 (24h): 18	*Ambystoma opacum*	Larvae	[34]
SC	Endrin	0.0002	0.0111	LC50 (96h): 18	*Ambystoma opacum*	Larvae	[34]
SC	Endrin	0.0001	0.0042	EC50 (24h): 48	*Ambystoma maculatum*	Larvae	[34]
SC	Endrin	0.0001	0.0036	LC50 (96h): 56	*Ambystoma maculatum*	Larvae	[34]
SC	Endrin	**2.00**	**100**	LC50 (96h): 0.002	*Pimephales promelas*	n.i.	[7]
SC	Endrin	0.0055	0.2740	LC50 (96h): 0.73	*Oncorhynchus mykiss*	n.i.	[5]
SC	Endrin	0.0333	**1.67**	NOEC (21d): 0.12	*Cyprinodon variegatus*	n.i.	[5]
P	Glyphosate	0.0027	0.0118	LC50 (7d): 23660	*Azolla caroliniana*	n.i.	[26]
P	Glyphosate	0.0054	0.0231	EC50 (<10d): 12100	Nonvascular Plants	n.i.	[6]
P	Glyphosate	0.0055	0.0235	EC50 (<10d): 11900	Vascular plants	n.i.	[6]
P	Glyphosate	0.0034	0.0147	EC50 (72h): 19000	Algae	n.i.	[7]
P	Glyphosate	0.0054	0.0233	EC50 (72h)-growth: 12000	*Raphidocelis subcapitata*	n.i.	[5]
P	Glyphosate	**5.42**	**23.33**	EC50 (7d): 12	*Lemna gibba*	n.i.	[5]
PC	Glyphosate	0.0012	0.0053	EC50 or LC50 (48 or 96h): 53200	Invertebrates	n.i.	[6]
PC	Glyphosate	0.0013	0.0056	NOAEC: 49900	Invertebrates	n.i.	[6]
PC	Glyphosate	0.0013	0.0056	LC50 (48h): 50339.42	*Daphnia*	n.i.	[7]
PC	Glyphosate	0.0007	0.0028	EC50 (48h): >100000	*Daphnia magna*	n.i.	[5]
PC	Glyphosate	0.0052	0.0224	NOEC (21d): 12500	*Daphnia magna*	n.i.	[5]
PC	Glyphosate	0.0016	0.0070	LC50 (96h): 40000	*Americamysis bahia*	n.i.	[5]
SC	Glyphosate	0.0015	0.0065	LC50 (96h): 43000	Fish	n.i.	[6]
SC	Glyphosate	0.0025	0.0109	NOAEC: 25700	Fish	n.i.	[6]
SC	Glyphosate	0.0003	0.0012	LC50 (96h): 227829.43	*Pimephales promelas*	n.i.	[7]
SC	Glyphosate	0.0007	0.0028	LC50 (96h): >100000	*Oncorhynchus mykiss*	n.i.	[5]

Trophic level	Pesticide	RQ Class 1/2	RQ Class 3	Endpoint: concentration (µg/L)	Tested organism	Life stage	Reference
SC	Glyphosate	0.0650	0.2800	NOEC (21d): 1000	*Danio rerio*	n.i.	[5]
SC	Glyphosate	0.0089	0.0384	LC50 (96h): 7300	*Rhamdia quelen*	Fingerling	[22]
P	Gution	0.000001	0.000001	EC50 (72h)-growth: 7150	*Raphidocelis subcapitata*	n.i.	[5]
PC	Gution	0.0045	0.0045	EC50 (48h): 1.1	*Daphnia magna*	n.i.	[5]
PC	Gution	0.0125	0.0125	LOEC (21d): 0.4	*Daphnia magna*	n.i.	[5]
PC	Gution	0.0227	0.0227	LC50 (96h): 0.22	*Americamysis bahia*	n.i.	[5]
SC	Gution	0.00001	0.00001	LC50 (96h): >890	*Xenopus laevis*	Embryo	[35]
SC	Gution	0.00001	0.00001	EC50 (96h)-malformed survivors: >890	*Xenopus laevis*	Embryo	[35]
SC	Gution	0.00001	0.00001	NOAEL (96h)-lenght: 480	*Xenopus laevis*	Embryo	[35]
SC	Gution	0.00000	0.00000	LOAEL (96h)-lenght: 1300	*Xenopus laevis*	Embryo	[35]
SC	Gution	0.00001	0.00001	NOAEL (96h)-deformity: 510	*Xenopus laevis*	Embryo	[35]
SC	Gution	0.00001	0.00001	LOAEL (96h)-deformity: <990	*Xenopus laevis*	Embryo	[35]
SC	Gution	0.000004	0.000004	NOAEL (96h)-mortality: 1300	*Xenopus laevis*	Embryo	[35]
SC	Gution	0.000001	0.000001	LOAEL (96h)-mortality: 3800	*Xenopus laevis*	Embryo	[35]
SC	Gution	0.000003	0.000003	LC50 (96h): 1470	*Pseudacris regilla*	Tadpole	[36]
SC	Gution	0.00003	0.00003	LOAEL (10d): 170	*Pseudacris regilla*	Tadpole	[36]
SC	Gution	0.0001	0.0001	NOAEL (10d): 70	*Pseudacris regilla*	Tadpole	[36]
SC	Gution	0.000003	0.000003	LC50 (96h): 1670	*Ambystoma gracile*	Larvae	[36]
SC	Gution	0.00002	0.00002	LOAEL (10d): 220	*Ambystoma gracile*	Larvae	[36]
SC	Gution	0.0001	0.0001	NOAEL (10d): 100	*Ambystoma gracile*	Larvae	[36]
SC	Gution	0.000003	0.000003	LC50 (96h): 1900	*Ambystoma maculatum*	Larvae	[36]
SC	Gution	0.00005	0.00005	LOAEL (10d): 110	*Ambystoma maculatum*	Larvae	[36]
SC	Gution	0.0002	0.0002	NOAEL (10d): 30	*Ambystoma maculatum*	Larvae	[36]
SC	Gution	0.0003	0.0003	LC50 (96h): 20	*Oncorhynchus mykiss*	n.i.	[5]
SC	Gution	0.0294	0.0294	NOEC (21d): 0.17	*Pimephales promelas*	n.i.	[5]
P	Heptachlor + epoxide	0.0000001/ 0.00004	0.0001	EC50 (72h): 264.29	Algae	n.i.	[7]

Trophic level	Pesticide	RQ Class 1/2	RQ Class 3	Endpoint: concentration (µg/L)	Tested organism	Life stage	Reference
P	Heptachlor	0.000001/ 0.0004	0.0011	EC50 (72h)-growth: 27	*Raphidocelis subcapitata*	n.i.	[5]
PC	Heptachlor	0.000001/ 0.0001	0.0004	LC50 (48h): 69.67	*Daphnia*	n.i.	[7]
PC	Heptachlor	0.000001/ 0.0002	0.0007	EC50 (48h): 42	*Daphnia magna*	n.i.	[5]
SC	Heptachlor	0.000001/ 0.0002	0.0005	LC50 (96h): 62.49	*Pimephales promelas*	n.i.	[7]
SC	Heptachlor	0.00001/ 0.0014	0.0043	LC50 (96h): 7	*Oncorhynchus mykiss*	n.i.	[5]
SC	Heptachlor	0.00004/ 0.01	0.0300	NOEC-growth: 1	*Danio rerio*	Juvenile	[12]
SC	Heptachlor	0.000004/ 0.001	0.0030	OEC-growth: 10	*Danio rerio*	Juvenile	[12]
P	Heptachlor epoxide	0.0000001/ 0.00002	0.0001	EC50 (72h): 478.39	Algae	n.i.	[7]
P	Heptachlor epoxide	0.0000000002/ 0.0000001	0.0000002	EC50 (72h)-growth: 200000	*Raphidocelis subcapitata*	n.i.	[5]
PC	Heptachlor epoxide	0.0000001/ 0.00003	0.0001	LC50 (48h): 299.43	*Daphnia*	n.i.	[7]
PC	Heptachlor epoxide	0.0000002/ 0.00004	0.0001	EC50 (48h): 240	*Daphnia magna*	n.i.	[5]
SC	Heptachlor epoxide	0.000004/ 0.001	0.0030	LC50 (96h): 10.09	*Pimephales promelas*	n.i.	[7]
SC	Heptachlor epoxide	0.000002/ 0.0005	0.0015	LC50 (96h): 20	*Oncorhynchus mykiss*	n.i.	[5]
P	Hexachlorobenzene	0.000001/ 0.00002	-	EC50 (72h): 263.79	Algae	n.i.	[7]

Trophic level	Pesticide	RQ Class 1/2	RQ Class 3	Endpoint: concentration (µg/L)	Tested organism	Life stage	Reference
P	Hexachlorobenzene	0.00003/ 0.0006	-	EC50 (72h)-growth: 10	*Scenedesmus abundans*	n.i.	[5]
PC	Hexachlorobenzene	0.00004/ 0.0008	-	LC50 (48h): 7.79	*Daphnia*	n.i.	[7]
PC	Hexachlorobenzene	0.0000006/ 0.00001	-	EC50 (24h): 500	*Daphnia magna*	n.i.	[5]
PC	Hexachlorobenzene	0.00009/ 0.002	-	NOEC (21d): >3	*Daphnia magna*	n.i.	[5]
SC	Hexachlorobenzene	0.000003/ 0.00007	-	LC50 (96h): 92.72	*Pimephales promelas*	n.i.	[7]
SC	Hexachlorobenzene	0.000009/ 0.002	-	LC50 (96h): 30	*Oncorhynchus mykiss*	n.i.	[5]
SC	Hexachlorobenzene	0.00006/ 0.001	-	NOEC (32d): >4.8	*Pimephales promelas*	n.i.	[5]
P	Lindane	0.0007	0.0741	EC50 (7d)-biomass: 27	*Lemna gibba*	n.i.	[5]
P	Lindane	0.000004	0.0004	EC50 (72h): 4982.09	Algae	n.i.	[7]
P	Lindane	0.00001	0.0008	EC50 (72h)-growth: 2500	*Scenedesmus abundans*	n.i.	[5]
PC	Lindane	0.0200	**2.00**	EC50 or LC50 (48 or 96h): 1	Invertebrates	n.i.	[6]
PC	Lindane	0.0004	0.0370	NOAEC: 54	Invertebrates	n.i.	[6]
PC	Lindane	0.0021	0.2137	LC50 (96h): 9.36	*Neocaridina denticulata*	Juvenile	[23]
PC	Lindane	0.00002	0.0016	LC50 (48h): 1257.95	*Daphnia*	n.i.	[7]
PC	Lindane	0.00001	0.0013	EC50 (48h): 1600	*Daphnia magna*	n.i.	[5]
PC	Lindane	0.0000004	0.00004	NOEC (21d): 54000	*Daphnia magna*	n.i.	[5]
PC	Lindane	0.0032	0.3175	LC50 (96h): 6.3	*Americamysis bahia*	n.i.	[5]
SC	Lindane	0.0118	**1.18**	LC50 (96h): 1.7	Fish	n.i.	[6]
SC	Lindane	0.0069	0.6897	NOAEC: 2.9	Fish	n.i.	[6]
SC	Lindane	0.0002	0.0236	LC50 (96h): 84.83	*Pimephales promelas*	n.i.	[7]
SC	Lindane	0.0069	**0.69**	LC50 (96h): 2.9	*Oncorhynchus mykiss*	n.i.	[5]
SC	Lindane	0.00001	0.0007	NOEC (21d): 2900	*Oncorhynchus mykiss*	n.i.	[5]

Trophic level	Pesticide	RQ Class 1/2	RQ Class 3	Endpoint: concentration (µg/L)	Tested organism	Life stage	Reference
P	Malathion	0.00001	0.0077	EC50 (72h)-growth: 13000	*Raphidocelis subcapitata*	n.i.	[5]
P	Malathion	0.00005	0.0490	EC50 (<10d): 2040	Nonvascular Plants	n.i.	[6]
P	Malathion	0.000004	0.0042	EC50 (<10d): 24000	Vascular plants	n.i.	[6]
P	Malathion	0.00002	0.0157	EC50 (72h): 6371.78	Algae	n.i.	[7]
PC	Malathion	**1.02**	**1020.4**	EC50 or LC50 (48 or 96h): 0.098	Invertebrates	n.i.	[6]
PC	Malathion	**1.67**	**1666.7**	NOAEC: 0.06	Invertebrates	n.i.	[6]
PC	Malathion	0.1111	**111.11**	LC50 (48h): 0.9	*Daphnia magna*	Neonate	[37]
PC	Malathion	0.0049	**4.92**	LC50 (48h): 20.32	*Daphnia*	n.i.	[7]
PC	Malathion	0.1429	**142.86**	EC50 (48h): 0.7	*Daphnia magna*	n.i.	[5]
PC	Malathion	**1.67**	**1666.67**	NOEC (21d): 0.06	*Daphnia magna*	n.i.	[5]
PC	Malathion	0.0667	**66.67**	LC50 (96h): 1.5	*Americamysis bahia*	n.i.	[5]
SC	Malathion	0.0244	**24.39**	LC50 (96h): 4.1	Fish	n.i.	[6]
SC	Malathion	0.0116	**11.63**	NOAEC: 8.6	Fish	n.i.	[6]
PC	Malathion	**3125**	**3125000**	LC50 (96h): 0.000032	*Chironomus ramosus*	Larvae	[32]
SC	Malathion	0.0223	**22.32**	LC50 (96h): 4.48	*Pimephales promelas*	n.i.	[7]
SC	Malathion	0.0056	**5.56**	LC50 (96h): 18	*Oncorhynchus mykiss*	n.i.	[5]
SC	Malathion	0.0011	**1.10**	NOEC (21d): 91	*Oncorhynchus mykiss*	n.i.	[5]
P	Metolachlor	0.2257	-	EC50 (72h): 44.3	*Raphidocelis subcapitata*	n.i.	[3]
P	Metolachlor	**1.25**	-	EC50 (<10d): 8	Nonvascular Plants	n.i.	[6]
P	Metolachlor	0.4762	-	EC50 (<10d): 21	Vascular plants	n.i.	[6]
P	Metolachlor	0.1190	-	EC50 (96h): 84	*Raphidocelis subcapitata*	n.i.	[4]
P	Metolachlor	0.0493	-	EC50 (96h): 203	*Chlorella vulgaris*	n.i.	[4]
P	Metolachlor	0.0088	-	EC50 (96h): 1138	*Chlamydomonas reinhardi*	n.i.	[4]
P	Metolachlor	0.0033	-	EC50 (96h): >3000	*Scenedesmus quadricauda*	n.i.	[4]
P	Metolachlor	0.0033	-	EC50 (96h): >3000	*Microcystis sp.*	n.i.	[4]
P	Metolachlor	0.0033	-	EC50 (96h): >3000	*Anabaena flosque*	n.i.	[4]
P	Metolachlor	0.0278	-	EC50 (14d): 360	*Lemna minor*	n.i.	[4]

Trophic level	Pesticide	RQ Class 1/2	RQ Class 3	Endpoint: concentration (µg/L)	Tested organism	Life stage	Reference
P	Metolachlor	0.1429	-	EC50 (14d): 70	*Ceratophyllum demersum*	n.i.	[4]
P	Metolachlor	0.0042	-	EC50 (14d): 2355	*Elodea canadensis*	n.i.	[4]
P	Metolachlor	0.0033	-	EC50 (14d): >3000	*Myriophyllum heterophyllum*	n.i.	[4]
P	Metolachlor	0.0413	-	EC50 (14d): 242	*Najas sp.*	n.i.	[4]
P	Metolachlor	0.0057	-	EC50 (24h)-growth rate: 1747	*Lemna minor*	n.i.	[27]
P	Metolachlor	0.2222	-	NOEC-growth rate: 45	*Lemna minor*	n.i.	[27]
P	Metolachlor	0.0056	-	EC50 (72h): 1798.98	Algae	n.i.	[7]
P	Metolachlor	0.0002	-	EC50 (72h)-growth: >57100	*Raphidocelis subcapitata*	n.i.	[5]
P	Metolachlor	0.2326	-	EC50 (7d)-biomass: >43	*Lemna gibba*	n.i.	[5]
P	Metolachlor	0.0033	-	NOEC (96h)-growth: 3000	*Anabaena sp.*	n.i.	[5]
PC	Metolachlor	0.0091	-	EC50 or LC50 (48 or 96h): 1100	Invertebrates	n.i.	[6]
PC	Metolachlor	**10**	-	NOAEC: 1	Invertebrates	n.i.	[6]
PC	Metolachlor	0.0004	-	EC50 (48h): 22300	*Daphnia magna*	Neonate	[3]
PC	Metolachlor	0.0034	-	EC50 (48h): <2900	*Hyalella azteca*	Juvenile	[8]
PC	Metolachlor	0.0004	-	EC50 (48h): 23000	*Ceriodaphnia dubia*	Juvenile	[8]
PC	Metolachlor	0.0002	-	EC50 (48h): 59000	*Daphnia magna*	Juvenile	[8]
PC	Metolachlor	0.0011	-	EC50 (96h): 9000	*Physa gyrina*	Juvenile	[8]
PC	Metolachlor	0.0010	-	EC50 (96h): 10000	*Lymnaea stagnalis*	Juvenile	[8]
PC	Metolachlor	0.0005	-	EC50 (24h): 21700	*Thamnocephalus platyurus*	Nauplii or early-instar juvenile	[8]
PC	Metolachlor	0.0007	-	LC50 (48h): 14740.14	*Daphnia*	n.i.	[7]
PC	Metolachlor	0.0004	-	EC50 (48h): >23500	*Daphnia magna*	n.i.	[5]
PC	Metolachlor	0.0141	-	LOEC (21d): >707	*Daphnia magna*	n.i.	[5]
PC	Metolachlor	0.0024	-	LC50 (96h): 4200	*Americamysis bahia*	n.i.	[5]
SC	Metolachlor	0.0026	-	LC50 (96h): 3800	Fish	n.i.	[6]
SC	Metolachlor	0.3333	-	NOAEC: 30	Fish	n.i.	[6]
SC	Metolachlor	0.0014	-	LC50 (96h): 7327.98	*Pimephales promelas*	n.i.	[7]

Trophic level	Pesticide	RQ Class 1/2	RQ Class 3	Endpoint: concentration (µg/L)	Tested organism	Life stage	Reference
SC	Metolachlor	0.0026	-	LC50 (96h): >3900	*Oncorhynchus mykiss*	n.i.	[5]
SC	Metolachlor	0.0100	-	NOEC (21d): 1000	*Cyprinodon variegatus*	n.i.	[5]
P	Metoxichlor	0.0001	0.0333	EC50 (72h)-growth: 600	*Scenedesmus quadricauda*	n.i.	[5]
P	Metoxichlor	0.0001	0.0336	EC50 (72h): 594.82	Algae	n.i.	[7]
PC	Metoxichlor	0.0010	**0.67**	LC50 (48h): 30	*Daphnia*	n.i.	[7]
PC	Metoxichlor	0.0214	**14.29**	EC50 or LC50 (48 or 96h): 1.4	Invertebrates	n.i.	[6]
PC	Metoxichlor	0.0385	**25.64**	EC50 (48h): 0.78	*Daphnia magna*	n.i.	[5]
PC	Metoxichlor	0.0300	**20**	NOEC (21d): 1	*Daphnia magna*	n.i.	[5]
SC	Metoxichlor	0.0002	0.1246	LC50 (96h): 160.55	*Pimephales promelas*	n.i.	[7]
SC	Metoxichlor	0.0020	**1.33**	LC50 (96h): 15	Fish	n.i.	[6]
SC	Metoxichlor	0.0006	0.3846	LC50 (96h): 52	*Oncorhynchus mykiss*	n.i.	[5]
P	Parathion	0.000004	0.0035	EC50 (72h)-growth: 10000	*Scenedesmus subspicatus*	n.i.	[5]
P	Parathion	0.00004	0.0354	EC50 (72h): 987.58	Algae	n.i.	[7]
PC	Parathion	0.1053	**92.11**	LC50 (48h): 0.38	*Daphnia magna*	Neonate	[37]
PC	Parathion	0.0533	**46.67**	LC50 (48h): 0.75	*Daphnia*	n.i.	[7]
PC	Parathion	0.0160	**14.00**	EC50 (48h): 2.5	*Daphnia magna*	n.i.	[5]
PC	Parathion	0.4000	**350.00**	NOEC (21d): 0.1	*Daphnia magna*	n.i.	[5]
PC	Parathion	0.3636	**318.18**	LC50 (96h): 0.11	*Americamysis bahia*	n.i.	[5]
SC	Parathion	0.00001	0.0054	LC50 (96h): 6500	*Carassius auratus*	Juvenile	[38]
SC	Parathion	0.00002	0.0140	NOEC: 2500	*Carassius auratus*	Juvenile	[38]
SC	Parathion	0.00005	0.0414	LC50 (96h): 845.4	*Pimephales promelas*	n.i.	[7]
SC	Parathion	0.00003	0.0233	LC50 (96h): 1500	*Oncorhynchus mykiss*	n.i.	[5]
SC	Parathion	0.0004	0.3571	NOEC (21d): >98	*Danio rerio*	n.i.	[4]
P	Pentachlorophenol	0.0375/ 0.1125	0.1125	EC50 (72h)-growth: 80	*Scenedesmus quadricauda*	n.i.	[4]
P	Pentachlorophenol	0.025/ 0.075	0.0750	EC50 (7d)-biomass: 120	*Lemna gibba*	n.i.	[4]
P	Pentachlorophenol	0.0075/	0.0225	NOEC (96h)-growth: >400	*Chlorella pyrenoidosa*	n.i.	[4]

Trophic level	Pesticide	RQ Class 1/2	RQ Class 3	Endpoint: concentration (μg/L)	Tested organism	Life stage	Reference
		0.0225					
P	Pentachlorophenol	0.005/ 0.016	0.0161	EC50 (72h): 559.24	Algae	n.i.	[6]
PC	Pentachlorophenol	0.06/ 0.18	0.1800	EC50 or LC50 (48 or 96h): 50	Invertebrates	n.i.	[5]
PC	Pentachlorophenol	0.006/ 0.02	0.0200	EC50 (48h): >450	*Daphnia magna*	n.i.	[4]
PC	Pentachlorophenol	0.0167/ 0.05	0.0500	NOEC (21d): >180	*Daphnia magna*	n.i.	[4]
PC	Pentachlorophenol	0.0059/ 0.017	0.0176	LC50 (48h): 510.12	*Daphnia*	n.i.	[6]
SC	Pentachlorophenol	0.0316/ 0.0947	0.0947	LC50 (96h): 95	Fish	n.i.	[5]
SC	Pentachlorophenol	0.005/ 0.0155	0.0155	LC50 (96h): 580	*Heteropneustes fossilis*	n.i.	[28]
SC	Pentachlorophenol	0.0047/ 0.0141	0.0141	LC50 (96h): 640	*Clarias batrachus*	n.i.	[28]
SC	Pentachlorophenol	0.0039/ 0.0117	0.0117	LC50 (96h): 770	*Channa punctatus*	n.i.	[28]
SC	Pentachlorophenol	0.00003/ 0.0001	0.0001	LC50 (48h): 98000	*Culex pipiens fatigans*	Larvae	[28]
SC	Pentachlorophenol	0.0167/ 0.05	0.0500	LC50 (96h): 180.09	*Pimephales promelas*	n.i.	[6]
SC	Pentachlorophenol	0.0176/ 0.0529	0.0529	LC50 (96h): >170	*Oncorhynchus mykiss*	n.i.	[4]
SC	Pentachlorophenol	0.06/ 0.18	0.1800	NOEC (21d): 50	*Carassius auratus*	n.i.	[4]
P	Simazine	0.0067	-	EC50 (72h)-biomass: 300	*Lemna gibba*	n.i.	[4]
P	Simazine	0.3333	-	EC50 (<10d): 6	Nonvascular Plants	n.i.	[5]
P	Simazine	0.0299	-	EC50 (<10d): 67	Vascular plants	n.i.	[5]
P	Simazine	0.0050	-	EC50 (72h)-growth: 400	*Scenedesmus subspicatus*	n.i.	[4]

Trophic level	Pesticide	RQ Class 1/2	RQ Class 3	Endpoint: concentration (µg/L)	Tested organism	Life stage	Reference
P	Simazine	0.0226	-	EC50 (72h): 88.6	Algae	n.i.	[6]
PC	Simazine	0.0020	-	EC50 or LC50 (48 or 96h): 1000	Invertebrates	n.i.	[5]
PC	Simazine	0.0500	-	NOAEC: 40	Invertebrates	n.i.	[5]
PC	Simazine	0.0001	-	LC50 (96h): 30600	*Pacifastacus leniusculus*	Juvenile	[19]
PC	Simazine	0.0000	-	LC50 (48h): 92100	*Daphnia pulex*	Adult	[38]
PC	Simazine	0.0001	-	LC50 (48h): 34803.17	*Daphnia*	n.i.	[6]
PC	Simazine	0.0018	-	EC50 (48h): 1100	*Daphnia magna*	n.i.	[4]
PC	Simazine	0.0008	-	LOEC (21d): 2500	*Daphnia magna*	n.i.	[4]
SC	Simazine	0.0003	-	LC50 (96h): 6400	Fish	n.i.	[5]
SC	Simazine	0.0333	-	NOAEC: 60	Fish	n.i.	[5]
SC	Simazine	0.0003	-	LC50 (96h): 7550	*Silurana tropicalis*	Tadpole	[20]
SC	Simazine	0.0000	-	LC50 (96h): 90000	*Lepomis macrochirus*	n.i.	[4]
SC	Simazine	0.0001	-	LC50 (96h): 24740.29	*Pimephales promelas*	n.i.	[6]
P	2.4.5–T	0.0010	0.0010	EC50 (7d)-growth: 2000	Green algae	n.i.	[4]
P	2.4.5–T	0.0003	0.0003	EC50 (72h): 6279.53	Algae	n.i.	[6]
PC	2.4.5–T	0.0004	0.0004	EC50 (48h): 5000	*Daphnia magna*	n.i.	[4]
PC	2.4.5–T	0.0002	0.0002	LC50 (48h): 10184.21	*Daphnia*	n.i.	[6]
SC	2.4.5–T	0.0015	0.0015	LC50 (96h): 1300	*Oncorhynchus mykiss*	n.i.	[4]
SC	2.4.5–T	0.0002	0.0002	LC50 (96h): 9189.71	*Pimephales promelas*	n.i.	[6]
P	Toxaphene	0.000001/ 0.00004	0.0008	EC50 (72h): 273.08	Algae	n.i.	[6]
PC	Toxaphene	0.000004/ 0.0002	0.0033	LC50 (48h): 63.51	*Daphnia*	n.i.	[6]
SC	Toxaphene	0.000001/ 0.0001	0.0011	EC50 (24h): 193	*Rana sphenocephala*	Larvae	[33]
SC	Toxaphene	0.000002/ 0.0001	0.0016	LC50 (96h): 130	*Rana sphenocephala*	Larvae	[33]

Trophic level	Pesticide	RQ Class 1/2	RQ Class 3	Endpoint: concentration (μg/L)	Tested organism	Life stage	Reference
SC	Toxaphene	0.0000009/ 0.00003	0.0007	EC50 (24h): 312	*Rana catesbeiana*	Larvae	[33]
SC	Toxaphene	0.000003/ 0.0001	0.0021	LC50 (96h): 99	*Rana catesbeiana*	Larvae	[33]
SC	Toxaphene	0.00001/ 0.0003	0.0058	EC50 (24h): 36	*Rana sylvatica*	Larvae	[33]
SC	Toxaphene	0.000001/ 0.0001	0.0011	LC50 (96h): 195	*Rana sylvatica*	Larvae	[33]
SC	Toxaphene	0.00001/ 0.0003	0.0055	EC50 (24h): 38	*Bufo americanus*	Larvae	[33]
SC	Toxaphene	0.00001/ 0.0003	0.0062	LC50 (96h): 34	*Bufo americanus*	Larvae	[33]
SC	Toxaphene	0.0000003/ 0.00001	0.0002	EC50 (24h): >1000	*Acris crepitans*	Larvae	[33]
SC	Toxaphene	0.000004/ 0.0001	0.0028	LC50 (96h): 76	*Acris crepitans*	Larvae	[33]
SC	Toxaphene	0.000002/ 0.0001	0.0012	EC50 (24h): 170	*Ambystoma opacum*	Larvae	[33]
SC	Toxaphene	0.000001/ 0.00003	0.0006	LC50 (96h): 342	*Ambystoma opacum*	Larvae	[33]
SC	Toxaphene	0.000001/ 0.00004	0.0009	EC50 (24h): 227	*Ambystoma maculatum*	Larvae	[33]
SC	Toxaphene	0.00001/ 0.0003	0.0062	LC50 (96h): 34	*Ambystoma maculatum*	Larvae	[33]
SC	Toxaphene	0.000001/ 0.00002	0.0005	LC50 (96h): 455.99	*Pimephales promelas*	n.i.	[6]
PC	2.4.5-TP	0.0001	0.0001	EC50 (48h): >140000	*Daphnia magna*	n.i.	[4]
PC	2.4.5-TP	0.0004	0.0004	LC50 (96h): >27900	*Americamysis bahia*	n.i.	[4]
SC	2.4.5-TP	0.0007	0.0007	LC50 (96h): >14800	*Oncorhynchus mykiss*	n.i.	[4]

Trophic level	Pesticide	RQ Class 1/2	RQ Class 3	Endpoint: concentration (µg/L)	Tested organism	Life stage	Reference
P	Trifluraline	0.0016	-	EC50 (72h): 124.77	Algae	n.i.	[6]
P	Trifluraline	0.0091	-	EC50 (<10d): 21.9	Nonvascular Plants	n.i.	[5]
P	Trifluraline	0.0040	-	EC50 (<10d): 49.7	Vascular plants	n.i.	[5]
PC	Trifluraline	0.0008	-	EC50 or LC50 (48 or 96h): 251	Invertebrates	n.i.	[5]
PC	Trifluraline	0.0833	-	NOAEC: 2.4	Invertebrates	n.i.	[5]
PC	Trifluraline	0.0008	-	EC50 (48h): 245	*Daphnia magna*	n.i.	[4]
PC	Trifluraline	0.0039	-	NOEC (21d): 51	*Daphnia magna*	n.i.	[4]
PC	Trifluraline	0.0027	-	LC50 (96h): 74	*Americamysis bahia*	n.i.	[4]
PC	Trifluraline	0.0004	-	LC50 (48h): 468.78	*Daphnia*	n.i.	[6]
SC	Trifluraline	0.0108	-	LC50 (96h): 18.5	Fish	n.i.	[5]
SC	Trifluraline	0.1053	-	NOAEC: 1.9	Fish	n.i.	[5]
SC	Trifluraline	0.0000	-	LC50 (120h): 9400	*Bombina bombina*	Embryo	[39]
SC	Trifluraline	0.0000	-	EC50 (120h): 5580	*Bombina bombina*	Embryo	[39]
SC	Trifluraline	0.0000	-	LC50 (120h): 11800	*Bombina bombina*	Larvae	[39]
SC	Trifluraline	0.0044	-	LC50 (96h): 45	*Cyprinus carpio*	6-month-old carp fry	[40]
SC	Trifluraline	0.0000	-	LC50 (96h): 9760	*Lithobates clamitans*	Tadpole	[41]
SC	Trifluraline	0.0013	-	LC50 (96h): 159.77	*Pimephales promelas*	n.i.	[6]
SC	Trifluraline	0.0023	-	LC50 (96h): 88	*Oncorhynchus mykiss*	n.i.	[4]
SC	Trifluraline	0.0000	-	NOEC (35d): 10000	*Pimephales promelas*	n.i.	[4]

d: day; h: hour; LC: lethal concentration; EC: effective concentration; LOEC: lowest observed effect concentration; LOAEL: lowest observable adverse effect level; NOAEC: no observed adverse effect concentration; NOEC: no observed effect concentration; NOAEL: observed adverse effect level; PNOEC: predicted no effect concentration; MATC: maximum acceptable toxicant concentrations; OEC: observed effect concentration. [a]Producer organism; [b]Primary consumer; [c]Secondary consumer.; [d]Demeton; [d1]Isomer S; [d2]Isomer O; [e] Degradation product of DDE; [f]Standards for bodies of water where there is fishing or cultivation of organisms for purposes of intensive consumption. All ecotoxicological studies were conducted in a laboratory setting, except for ref. [4-6] where this information was not available.

References

1. Brasil *Resolução CONAMA N° 357 de 18 de Março de 2005. Dispõe Sobre a Classificação Dos Corpos de Água e Diretrizes Ambientais Para o Seu Enquadramento, Bem Como Estabelece as Condições e Padrões de Lançamento de Efluentes, e Dá Outras Providências.*; Brasil, 2005; pp. 58–63;.

2. Souissi, Y.; Bouchonnet, S.; Bourcier, S.; Kusk, K.O.; Sablier, M.; Andersen, H.R. Identification and Ecotoxicity of Degradation Products of Chloroacetamide Herbicides from UV-Treatment of Water. *Science of the Total Environment* **2013**, *458–460*, 527–534, doi:10.1016/j.scitotenv.2013.04.064.

3. Fairchild, J.F.; Ruessler, D.S.; Carlson, A.R. Comparative Sensitivity of Five Species of Macrophytes and Six Species of Algae to Atrazine, Metribuzin, Alachlor, and Metolachlor. *Environ Toxicol Chem* **1998**, *17*, 1830–1834, doi:10.1002/etc.5620170924.

4. PPDB Pesticide Properties DataBase Available online: http://sitem.herts.ac.uk/aeru/ppdb/en/index.htm (accessed on 29 May 2022).

5. USEPA Aquatic Life Benchmarks and Ecological Risk Assessments for Registered Pesticides Available online: https://www.epa.gov/pesticide-science-and-assessing-pesticide-risks/aquatic-life-benchmarks-and-ecological-risk#ref_1 (accessed on 29 May 2022).

6. NORMAN NORMAN Ecotoxicology Database Available online: https://www.norman-network.com/nds/susdat/ (accessed on 29 May 2022).

7. Ivey, C.D.; Besser, J.M.; Ingersoll, C.G.; Wang, N.; Rogers, D.C.; Raimondo, S.; Bauer, C.R.; Hammer, E.J. Acute Sensitivity of the Vernal Pool Fairy Shrimp, Branchinecta Lynchi (Anostraca; Branchinectidae), and Surrogate Species to 10 Chemicals. *Environ Toxicol Chem* **2017**, *36*, 797–806, doi:10.1002/etc.3723.

8. Howe, G.E.; Gillis, R.; Mowbray, R.C. Effect of Chemical Synergy and Larval Stage on the Toxicity of Atrazine and Alchlor to Amphibian Larvae. *Environ Toxicol Chem* **1998**, *17*, 519–525, doi:https://doi.org/10.1002/etc.5620170324.

9. Peebua, P.; Kruatrachue, M.; Pokethitiyook, P.; Singhakaew, S. Histopathological Alterations of Nile Tilapia, Oreochromis Niloticus in Acute and Subchronic Alachlor Exposure. *J Environ Biol* **2008**, *29*, 325–331.

10. Huang, L.; Xi, Y.; Zha, C.; Wen, X. Responses in the Population Growth and Reproduction of Freshwater Rotifer Brachionus Calyciflorus to Four Organochlorine Pesticides. *Ann Limnol* **2013**, *49*, 79–85, doi:10.1051/limn/2013038.

11. Campagna, A.F.; Eler, M.N.; Fracácio, R.; Rodrigues, B.K.; Verani, N.F. The Toxic Potential of Aldrin and Heptachlor on Danio Rerio Juveniles (Cypriniformes, Cyprinidae). *Ecotoxicology* **2007**, *16*, 289–298, doi:10.1007/s10646-006-0127-8.

12. Satyanarayan, S.; Bejankiwar, R.S.; Chaudhari, P.R.; Kotangale, J.P.; Satyanarayan, A. Impact of Some Chlorinated Pesticides Onthe Haematology of the Cyprinus Carpio and Puntius Ticto. *Journal of Environmental Sciences* **2004**, *16*, 631–634.

13. Werner, I.; Nagel, R. Stress Proteins HSP60 and HSP70 in Three Species of Amphipods Exposed to Cadmium, Diazinon, Dieldrin and Fluoranthene. *Environ Toxicol Chem* **1997**, *16*, 2393–2403, doi:10.1002/etc.5620161127.

14. Schuytema, G.S.; Nebeker, A. v.; Griffis, W.L.; Wilson, K.N. Teratogenesis, Toxicity, and Bioconcentration in Frogs Exposed to Dieldrin. *Archives Environmental Contamination and Toxicology* **1991**, *21*, 332–350.

15. della Vechia, J.F.; Cruz, C.; Silva, A.F.; Cerveira, W.R.; Garlich, N. Macrophyte Bioassay Applications for Monitoring Pesticides in the Aquatic Environment. *Planta Daninha* **2016**, *34*, 597–603, doi:10.1590/S0100-83582016340300021.

16. Lu, Z.; Zhao, B.; Yang, J.; Snell, T.W. Effects of Atrazine and Carbaryl on Growth and Reproduction of the Rotifer Brachionus Calyciflorus Pallas. *J Freshw Ecol* **2012**, *27*, 527–537, doi:10.1080/02705060.2012.675758.

17. He, H.; Yu, J.; Chen, G.; Li, W.; He, J.; Li, H. Acute Toxicity of Butachlor and Atrazine to Freshwater Green Alga Scenedesmus Obliquus and Cladoceran Daphnia Carinata. *Ecotoxicol Environ Saf* **2012**, *80*, 91–96, doi:10.1016/j.ecoenv.2012.02.009.

18. Phyu, Y.L.; Warne, M.St.J.; Lim, R.P. Toxicity and Bioavailability of Atrazine and Molinate to the Freshwater Shrimp (Paratya Australiensis) under Laboratory and Simulated Field Conditions. *Ecotoxicol Environ Saf* **2005**, *60*, 113–122, doi:10.1016/j.ecoenv.2004.07.006.

19. Velisek, J.; Kouba, A.; Stara, A. Acute Toxicity of Triazine Pesticides to Juvenile Signal Crayfish (Pacifastacus Leniusculus). *Neuroendocrinology Letters* **2013**, *34*, 31–36.

20. Saka, M.; Tada, N.; Kamata, Y. Chronic Toxicity of 1,3,5-Triazine Herbicides in the Postembryonic Development of the Western Clawed Frog Silurana Tropicalis. *Ecotoxicol Environ Saf* **2018**, *147*, 373–381, doi:10.1016/j.ecoenv.2017.08.063.

21. KreutzI, L.C.; Barcellos, L.J.G.; Silva, T.O.; Anziliero, D.; Martins, D.; Lorenson, M.; Marteninghe, A.; Silva, L.B. Acute Toxicity Test of Agricultural Pesticides on Silver Catfish (Rhamdia Quelen) Fingerlings. *Ciência Rural* **2008**, *38*, 1050–1055.

22. Huang, D.J.; Chen, H.C. Effects of Chlordane and Lindane on Testosterone and Vitellogenin Levels in Green Neon Shrimp (Neocaridina Denticulata). *Int J Toxicol* **2004**, *23*, 91–95, doi:10.1080/10915810490435604.

23. Manar, R.; Vasseur, P.; Bessi, H. Chronic Toxicity of Chlordane to Daphnia Magna and Ceriodaphnia Dubia: A Comparative Study. *Environ Toxicol* **2012**, *27*, 90–97, doi:10.1002/tox.

24. Manar, R.; Bessi, H.; Vasseur, P. Reproductive Effects and Bioaccumulation of Chlordane in Daphnia Magna. *Environ Toxicol Chem* **2009**, *28*, 2150–2159, doi:10.1897/08-564.1.

25. Silva, A.F.; Cruz, C.; Neto, A.N.; Pitelli, R.A. Ecotoxicidade de Herbicidas Para a Macrófita Aquática (Azolla Caroliniana). *Planta Daninha* **2012**, *30*, 541–546, doi:https://doi.org/10.1590/S0100-83582012000300009.

26. Sanford, M.; Washuck, N.; Carr, K.; Prosser, R.S. Pulsed Exposure of the Macrophyte Lemna Minor to Herbicides and the Mayfly Neocloeon Triangulifer to Diamide Insecticides. *Chemosphere* **2021**, *273*, 1–7, doi:10.1016/j.chemosphere.2020.128582.

27. Silva Pinto, T.J.; Moreira, R.A.; Silva, L.C.M.; Yoshii, M.P.C.; Goulart, B.V.; Fraga, P.D.; Rolim, V.L.S.; Montagner, C.C.; Daam, M.A.; Espindola, E.L.G. Toxicity of Fipronil and 2,4-D Formulations (Alone and in a Mixture) to the Tropical Amphipod Hyalella Meinerti. *Environmental Science and Pollution Research* **2021**, *28*, 38308–38321, doi:10.1007/s11356-021-13296-9.

28. Farah, M.A.; Ateeq, B.; Ali, M.N.; Sabir, R.; Ahmad, W. Studies on Lethal Concentrations and Toxicity Stress of Some Xenobiotics on Aquatic Organisms. *Chemosphere* **2004**, *55*, 257–265, doi:10.1016/j.chemosphere.2003.10.063.

29. Holcombe, G.W.; Phipps, G.L.; Fiandt, J.T. Effects of Phenol, 2,4-Dimethylphenol, 2,4-Dichlorophenol, and Pentachlorophenol on Embryo, Larval, and Early-Juvenile Fathead Minnows (Pimephales Promelas). *Archives Environmental Contamination Toxicology* **1982**, *11*, 73–78.

30. Phipps, G.L.; Holcombe, G.W.; Fiandt, J.T. Acute Toxicity of Phenol and Substituted Phenols to the Fathead Minnow. *Bulletin Environmental Contamination and Toxicology* **1981**, *26*, 585–593.

31. Majumdar, T.N.; Gupta, A. Acute Toxicity of Endosulfan and Malathion on Chironomus Ramosus (Insecta : Diptera: Chironomidae) from North Cachar Hills, Assam, India. *J Environ Biol* **2009**, *30*, 469–470.

32. Carriger, J.F.; Hoang, T.C.; Rand, G.M.; Gardinali, P.R.; Castro, J. Acute Toxicity and Effects Analysis of Endosulfan Sulfate to Freshwater Fish Species. *Arch Environ Contam Toxicol* **2011**, *60*, 281–289, doi:10.1007/s00244-010-9623-1.

33. Hall, R.J.; Swineford, D.M. Acute Toxicities of Toxaphene and Endrin to Larvae of Seven Species of Amphibians. *Toxicol Lett* **1981**, *8*, 331–336.

34. Schuytema, G.S.; Nebeker, A. v.; Griffis, W.L. Toxicity of Guthion® and Guthion® 2S to Xenopus Laevis Embryos. *Archives Environmental Contaminations and Toxicology* **1994**, *27*, 250–255.

35. Nebeker, A. v; Schuytema, G.S.; Griffis, W.L.; Cataldo, A. Impact of Guthion on Survival and Growth of the Frog Pseudacris Regilla and the Salamanders Ambystoma Gracile and Ambystoma Maculatum. *Archives of Environmental Contamination andd Toxicology* **1998**, *35*, 48–51.

36. Ren, Z.; Zha, J.; Ma, M.; Wang, Z.; Gerhardt, A. The Early Warning of Aquatic Organophosphorus Pesticide Contamination by On-Line Monitoring Behavioral Changes of Daphnia Magna. *Environ Monit Assess* **2007**, *134*, 373–383, doi:10.1007/s10661-007-9629-y.

37. Ferrari, A.; Venturino, A.; D’Angelo, A.M.P. Time Course of Brain Cholinesterase Inhibition and Recovery Following Acute and Subacute Azinphosmethyl, Parathion and Carbaryl Exposure in the Goldfish (Carassius Auratus). *Ecotoxicol Environ Saf* **2004**, *57*, 420–425, doi:10.1016/S0147-6513(02)00069-6.

38. Fitzmayer, K.M.; Geiger, J.G.; Avyle, M.J. van den Effects of Chronic Exposure to Simazine on the Cladoceran, Daphnia Pulex. *Archives Environmental Contamination and Toxicology* **1982**, *11*, 603–609.

39. Sayim, F. Toxicity of Trifluralin on the Embryos and Larvae of the Red-Bellied Toad, Bombina Bombina. *Turkish Journal of Zoology* **2010**, *34*, 479–486, doi:10.3906/zoo-0811-21.

40. Poleksic, V.; Karan, V. Effects of Trifluralin on Carp: Biochemical and Histological Evaluation. *Ecotoxicol Environ Saf* **1999**, *43*, 213–221.

41. Weir, S.M.; Yu, S.; Salice, C.J. Acute Toxicity of Herbicide Formulations and Chronic Toxicity of Technical-Grade Trifluralin to Larval Green Frogs (Lithobates Clamitans). *Environ Toxicol Chem* **2012**, *31*, 2029–2034, doi:10.1002/etc.1910.
